# Circulating proteins reveal prior use of menopausal hormonal therapy and increased risk of breast cancer

**DOI:** 10.1016/j.tranon.2022.101339

**Published:** 2022-01-13

**Authors:** Cecilia E. Thomas, Leo Dahl, Sanna Byström, Yan Chen, Mathias Uhlén, Anders Mälarstig, Kamila Czene, Per Hall, Jochen M. Schwenk, Marike Gabrielson

**Affiliations:** a Science for Life Laboratory, Department of Protein Science School of Engineering Sciences in Chemistry, Biotechnology and Health, KTH Royal Institute of Technology, Tomtebodavägen 23, Solna, Stockholm 171 65, Sweden; b Department of Medical Epidemiology and Biostatistics, Karolinska Institutet Nobels väg 12A, Stockholm SE-171 77, Sweden; c Department of Medicine, Karolinska Institutet, Stockholm, Sweden; d Department of Oncology, Södersjukhuset, Stockholm, Sweden

**Keywords:** MHT, Menopausal Hormone Therapy, SBA, Suspension Bead Array, MJI, Mean Jaccard Index, FDR, False Discovery Rate, KARMA, Karolinska Mammography Project for Risk Prediction of Breast Cancer, MFI, Median Fluorescence Intensity, Breast cancer, Risk prediction, Plasma proteomics, Affinity proteomics, Karma cohort, Archetypal analysis, Clustering, Patient stratification, Menopausal hormonal therapy

## Abstract

Accessible risk predictors are crucial for improving the early detection and prognosis of breast cancer. Blood samples are widely available and contain proteins that provide important information about human health and disease, however, little is still known about the contribution of circulating proteins to breast cancer risk prediction. We profiled EDTA plasma samples collected before diagnosis from the Swedish KARMA breast cancer cohort to evaluate circulating proteins as molecular predictors. A data-driven analysis strategy was applied to the molecular phenotypes built on 700 circulating proteins to identify and annotate clusters of women. The unsupervised analysis of 183 future breast cancer cases and 366 age-matched controls revealed five stable clusters with distinct proteomic plasma profiles. Among these women, those in the most stable cluster (*N* = 19; mean Jaccard index: 0.70 ± 0.29) were significantly more likely to have used menopausal hormonal therapy (MHT), get a breast cancer diagnosis, and were older compared to the remaining clusters. The circulating proteins associated with this cluster (FDR < 0.001) represented physiological processes related to cell junctions (F11R, CLDN15, ITGAL), DNA repair (RBBP8), cell replication (TJP3), and included proteins found in female reproductive tissue (PTCH1, ZP4). Using a data-driven approach on plasma proteomics data revealed the potential long-lasting molecular effects of menopausal hormonal therapy (MHT) on the circulating proteome, even after women had ended their treatment. This provides valuable insights concerning proteomics efforts to identify molecular markers for breast cancer risk prediction.

## Highlights

• Current risk prediction models use a variety of factors to identify women at risk of developing breast cancer.
• Proteins circulating in blood represent an attractive but currently still underrepresented source of candidates serving as molecular risk factors.
• Plasma samples from women participating in a prospective breast cancer cohort study were studied for proteomic risk factors related to a future breast cancer diagnosis.
• Applying data-driven approaches on the levels of circulating proteins, women with future breast cancers and previous use of menopausal hormone therapy were identified.
• Menopausal hormone therapy was found to alter components of the circulating proteomes even years after the treatment ended.

## Introduction

Breast cancer is the most common cancer among females worldwide and the leading cause of cancer-related mortality in middle-aged women [1]. Improving risk prediction and early detection is crucial for providing a better prognosis and improving the chances of survival. Circulating biomarkers have a great potential for simple and minimally invasive health assessment. Although studies show promising results for blood tests detecting common cancers of the ovary, liver, stomach, pancreas, esophagus, colorectum, and lung by circulating proteins [2], identifying putative biomarkers for risk prediction and early detection of breast cancer has thus far been less successful [2], [3], [4]. One reason could be that many breast cancers are already being detected at an early stage in mammographic screening programs. Blood levels of early-stage cancer biomarkers are expected to be low [5], possibly even too low to detect before the tumor can be uncovered by mammography. Further complicating the search for biomarkers, breast cancer, like most cancers, does not represent a single homogeneous phenotype but consists of multiple subtypes, each arising from distinct molecular mechanisms and progressing on diverging clinical paths. So far, proteomic studies have suggested that plasma protein biomarkers for breast cancer may be both subtype and stage-specific [3,[6], [7], [8], [9]]. In addition, there is a growing awareness about inter-individual diversity of molecular profiles even across clinically healthy individuals [10]. Moreover, germline genetic variation may add another layer of complexity to finding circulating proteins as common disease biomarkers [11].

Phenotypic and molecular heterogeneity often limits the utility of classical dichotomous case-control analyses. These can prove challenging to delineate or simplistic for understanding the underlying molecular subtypes. In these instances, alternative strategies, such as unsupervised and data-driven methods, can allow for novel hypotheses and the finding of translational biomarkers. Our ambition is to yield unexpected patterns in the data to deliver subgroups that can then readily be linked to molecular phenotypes, clinical risk factors, and potentially stratified intervention. Machine learning-based clustering is one approach to achieve such explorative, data-driven subtyping. It has been applied successfully in other disease areas, such as diabetes [12] and heart failure [13]. Clustering approaches have also previously been applied to breast cancer for prognosis stratification [14,15] and tumor subtyping [14,16,17] using a variety of clinical and molecular parameters. We used data-driven clustering to stratify women by decomposing their molecular profiles as defined by circulating proteins and to study the resulting groups for breast cancer risk and risk factors.

With access to the Swedish prospective population-based KARMA (Karolinska Mammography Project for Risk Prediction of Breast Cancer) cohort [18,19], we applied exploratory profiling of circulating proteins using a multiplexed affinity proteomics approach based on antibody suspension bead array (SBA) assays. The method allows for many proteins to be screened in small plasma volumes of a large number of samples [20]. We used a data-driven clustering approach on the plasma proteomics data from age-matched breast cancer cases and controls collected before diagnosis to identify proteins associated with phenotypic traits and breast cancer risk factors. The emerging clusters were investigated for associations with clinical parameters, and proteins important for the clustering were identified. We assumed heterogeneity in breast cancer development and risk and wanted to learn how risk factors influence the plasma proteome. Our study aimed to improve our currently limited knowledge about using the circulating proteins to identify women at risk of developing breast cancer.

## Material and methods

### Study design, sample inclusion criteria, and data collection

**Fig. 1:**
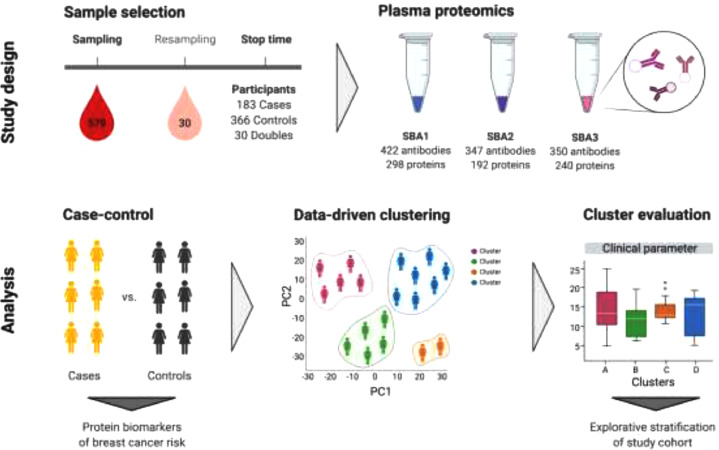
Overview of study design and data analysis. SBA; suspension bead array.

The source population was the KARMA (Karolinska Mammography Project for Risk Prediction of Breast Cancer) Cohort consisting of 70,877 women visiting any of four Swedish mammography units during 2011–2013 [18,19]. All participants signed informed consent forms before joining the KARMA study, and the ethical review board of Karolinska Institutet approved the study (DNR 2010/958–31/1). Cases were defined as women diagnosed with breast cancer (*N* = 183) after entering the cohort. Controls were 1:2 matched to each case based on age at last regular screening mammogram and study site (Fig. 1).

The median time from blood draw to breast cancer diagnosis was 23 days (range 0–588 days). Twelve of the incident cases had been diagnosed with breast cancer in the past (5–30 years before blood draw; median 11 years). For all, the previous breast tumor was in the other breast than the tumor that was detected after sampling. 2 controls had previous breast cancer diagnoses, 6 and 16 years before study entry.

In addition, 19 cases and 10 controls had been diagnosed with other types of cancer before sampling (cases: 0.6–35 years; controls: 1–46 years). An additional set of 60 samples from 30 cancer-free individuals from the KARMA cohort were included for quality control (named ‘doubles’). These 30 individuals were sampled on two separate occasions with a median time interval of 19.1 months (range 10.7–19.9) between sampling times.

Raw (unprocessed) digital mammograms for each study participant were collected at KARMA study enrolment as previously described [18,21]. Additional phenotypic information was obtained from the KARMA study questionnaire, and national health care registers [18]. BMI (body mass index) was calculated at study entry and was based on self-reported height and weight. Information on tumor characteristics was obtained by linkage to the Swedish nationwide cancer registry. Information on menopausal hormonal therapy (MHT) and statin use was extracted from the Swedish drug prescription registry and supplemented with data collected from KARMA questionnaires. Anatomical Therapeutic Chemical (ATC) codes were extracted for MHT prescriptions containing only estrogens, only progestogens, or a combination of estrogens and progestogens, as well as for the use of lipophilic and hydrophilic statins (**Supplementary Table S1**). MHT information was also available from the KARMA questionnaire. It was merged with the drug registry used for analysis. In instances where both registry and questionnaire data was available, registry data took precedence.

### Plasma sample collection

Non-fasting EDTA (ethylenediaminetetraacetic acid) plasma samples of peripheral blood were collected between January 2011 and September 2012 from the KARMA participants at the study enrolment [18]. All blood samples were handled following a strict 30-hours cold-chain protocol and were processed at the Karolinska Institutet high-throughput biobank in Stockholm, Sweden.

### Antibody bead arrays

We used antibody suspension bead arrays (SBA) to determine protein profiles in plasma samples. The SBAs were generated using carboxylated magnetic beads (MagPlex-C, Luminex Corp.) as previously described elsewhere in [20]. All plasma samples within each study set were retrieved from the biobank and analyzed at the same point in time. Plasma samples stored at −80 °C were thawed at 4 °C and randomized across seven 96-well microtiter plates in a stratified manner: Each double pair and trio (case and two matched controls) were placed within the same plate, resulting in an even distribution of cases, controls, and doubles across all seven plates. Samples were assayed in 384 well plates, where the fourth quadrant in each 384-well contained the same 96 samples that originated from one of the crude 96-well sample plates. In addition, all plates included four aliquot replicates from a crude plasma pool from all individuals included in the study. Samples were biotinylated, diluted, heat-treated at 56 °C, and combined with the bead array on two separate 384-well assay plates following previously described protocols [22]. The protein levels were reported as units of the median fluorescence intensity (MFI) from measuring at least 32 beads per antibody assay.

### Protein target selection

We used antibodies derived from the Human Protein Atlas [23] to construct three SBAs. The SBAs were built on sets of 422, 347, and 350 antibodies (SBA1-SBA3, **Supplementary Fig. S1**) as previously described in [10]. These targeted a total of 701 unique protein-encoding genes, and a complete list of all antibodies included in the study is provided in **Additional file 1**. The 422 antibodies included in the first bead array (SBA1) targeted 295 protein-encoding genes annotated to extracellular matrix (www.uniprot.org) [24], including integrins (*N* = 27), laminins (*N* = 21), matrix metalloproteases (*N* = 21), metallopeptidases (*N* = 18), and proteoglycans (*N* = 16). Most of the antibodies (82%) in SBA1 targeted secreted proteins. The 347 antibodies in SBA2 included 243 antibodies (127 proteins) targeting breast cancer-related proteins from literature, 62 antibodies towards 55 proteins with strong expression in breast tissue according to RNAseq data (www.proteinatlas.org), 39 antibodies towards 11 proteins with indicative associations to breast cancer from previous screenings and 3 controls. The 350 antibodies against 241 protein-encoding genes included in the third suspension bead array (SBA3) were selected based on possible relationship to mammographic breast density, cancer development and/or progression, tissue composition and/or remodeling. Due to overlap between the different arrays, the total number was 1073 unique antibodies targeting 701 unique proteins. This included sets of paired antibodies with common protein targets.

### Data processing

The generated raw protein profile data was normalized and annotated as follows. Antibody-specific probabilistic quotient normalization (Abs-PQN) [10] was applied per 96-well plate to reduce within-plate sample-to-sample variation. Between-plate normalization was performed using a multidimensional (MA) normalization method [25] (**Supplementary Fig. S2**).

A set of 96 duplicated samples was used to assess technical variation and to confirm the reproducibility of antibody profiles within all three SBAs. Prior to statistical analyses, the data were annotated based on assay performance using three criteria. Internal controls and antibodies were excluded from proceeding analyses if they showed low reproducibility in replicated analyses (as rho<0.7), correlation to human IgG levels (rho>0.5), or elevated background levels in assays with sample-free buffers (MFI_Empty_ > mean(MFI_Sample_) + 3 × sd(MFI_Sample_)). Replicated samples were also excluded before the analyses.

### Case-control analysis

For contrasting cases versus controls, conditional logistic regression models considering the age- and sampling location matching of cases and controls were applied to normalized, Ab-filtered, and log-transformed proteomics data. Three models were compared. In model 1, BMI and study entry date were included as exposure variables. Model 2 included exposure variables for absolute area-based breast density, postmenopausal status (yes/no), and MHT use (yes/no) in addition to BMI and entry date. In model 3, smoking (packs/year), alcohol (grams/week), and childbirth (yes/no) were included as exposure variables in addition to the variables in model 2. Due to missing values for BMI (4 missing), area-based density (20 missing), MHT usage (5 missing), smoking (3 missing), alcohol (2 missing), and childbirth (1 missing), 540 samples (181 cases, 359 controls) were analyzed in model 1, 490 samples (167 cases, 323 controls) were analyzed in model 2 and 484 (165 cases, 319 controls) were analyzed in model 3. Statistical modeling was performed using the ”clogit” function of the "survival" R package (version 3.1.8) [26,27].

### Unsupervised clustering

We performed an unsupervised archetype clustering of the proteomics data to identify clusters of individuals with similar protein profiles. These profiles were subsequently associated with clinical risk factors and other traits.

The quality-controlled proteomics data sets were linearly adjusted for BMI, entry date, and age at sampling. Clustering was performed using archetypal analysis where each participant can be described as a combination of archetypes representing extremes in the data. Archetypal analysis was performed using the “archetypal“ function of the “archetypal” R package (version 1.1.0) [28]. The "find_optimal_kappas" function of the package was used to determine the optimal number of archetypes where the benefit of using more archetypes is marginal **(Supplementary materials and methods, Unsupervised clustering).** After the archetypal analysis, each participant was assigned to the archetype they had the highest probability of belonging to. To validate the clusters, we tested the stability of the clusters when the data was changed slightly [29]. This was done by bootstrap analysis: A subset of patients was randomly selected and the results from clustering this subset was compared with the outcome when clustering the original data. For technical assessment of the clustering, the results of the archetypal analysis were used to predict the archetype coefficients of doubles and replicates that had been excluded from the original clustering. This was done using the "predict" function of the “stats” R package (version 3.6.0) on an "archetypes" object of the "archetypes" R package (version 2.2.0.1) [30]. Further details on the clustering analysis can be found in the supplementary material. The protein profiles of the resulting clusters were visualized in a heatmap using the R packages "ComplexHeatmap" (version 2.2.0, [31]) and "circlize" (version 0.4.13, [32]).

### Statistical tests of cluster characteristics

We compared the clusters to investigate how the differences in protein levels driving the clustering materialized at the clinical level. Similarly, we compared the genetic predisposition to breast cancer to assess if the differences in protein levels might be genetically driven. Details on the genetic data and calculation of polygenic risk scores (PRSs) are given in the supplementary material **(Supplementary materials and methods, Genotyping).** The Wilcoxon rank-sum test was used for continuous variables and Fisher's exact test for categorical variables. Testing of the influence of potential genetic components between the clusters was done by the absolute values of PRS in the clusters as a continuous variable. All *P-*values were two-sided and considered statistically significant if <0.05.

To rank the proteins driving a cluster, we first performed differential abundance analysis comparing a cluster to the remaining samples using a two-tailed *t*-test. The resulting *p*-values were corrected for multiple comparisons using Benjamini-Hochberg adjustment, resulting in false discovery rates (FDRs) for each protein. To shortlist representative proteins for a cluster, we selected the union of those with the lowest p-values and the highest (positive or negative) difference in relative abundance.

Next, we performed pathway analysis to summarize the potential functions of differentially abundant circulating proteins. We began by applying Over-Representation Analysis (ORA) using two criteria for protein selection; proteins with an FDR < 0.05 and the top 50 proteins with the lowest p-value, using the “gost” function of the “gprofiler2” R package (version 0.1.8) [33]. Next, we applied Gene Set Enrichment Analysis (GSEA), where all proteins were included but ranked by their p-value and direction of differential abundance, using the “fgsea” R package (version 1.12.0) from Bioconductor [34].

The levels of the selected proteins in all participants were associated with the variables dense area (adjusted for BMI and age) and MHT status (never taken, taken before study entry, taking at entry) using linear and logistic regression, respectively. All data handling and statistical analyses were performed in R version 3.6.0.

## Results

### Characterizing the cohort

The selected study population consisted of 183 cases and 366 matched controls (Table 1), as well as 30 doubles that were sampled twice over time (**Supplementary Table S2**). Cases and controls had similar BMI, but cases had a higher absolute area-based breast density (*p* = 0.0045). 74.9% of cases were postmenopausal, with similar proportions for controls. 48.1% of cases and 46.7% of controls had never taken MHT, with similar numbers for statin use. Most of the tumors were positive for ER (74.9%) and PR (59.6%), only a few confirmed HER2 positive (7.7%). More than half of the tumors were invasive (54.1%) with histological grade ≥2 (76.5%) but without lymph node invasion (78.1%). Women were recruited at four centers, but no differences between sampling centers were observed at the protein level (**Supplementary Fig. S3**).

**Table 1:** Overview of clinical characteristics for cases and controls, and tumor characteristics for cases. P-values are from comparing cases and controls using Wilcoxon rank-sum tests for continuous variables and Fisher's exact tests for categorical variables.

	**Total (*N*** **=** **549)**	**Cases (*N*** **=** **183)**	**Controls (*N*** **=** **366)**	**P-value**
**Age**				
Mean (SD)	59.6 (9.28)	59.6 (9.30)	59.6 (9.28)	1
Median [Min, Max]	62.0 [39.0, 81.0]	62.0 [39.0, 81.0]	62.0 [39.0, 81.0]	
**BMI**				
Mean (SD)	25.6 (4.19)	25.8 (3.78)	25.5 (4.38)	0.13
Median [Min, Max]	24.9 [17.6, 49.0]	25.4 [18.5, 39.2]	24.7 [17.6, 49.0]	
Missing	4 (0.7%)	1 (0.5%)	3 (0.8%)	
**Sampling center**				
Helsingborg Hospital	283 (51.5%)	95 (51.9%)	188 (51.4%)	0.99
Landskrona Hospital	23 (4.2%)	7 (3.8%)	16 (4.4%)	
Skåne University Hospital, Lund	20 (3.6%)	7 (3.8%)	13 (3.6%)	
Stockholm South General Hospital	223 (40.6%)	74 (40.4%)	149 (40.7%)	
**Menopausal status**				
Premenopausal	130 (23.7%)	45 (24.6%)	85 (23.2%)	0.75
Postmenopausal	418 (76.1%)	137 (74.9%)	281 (76.8%)	
Missing	1 (0.2%)	1 (0.5%)	0 (0%)	
**Dense area (cm2)**				
Mean (SD)	27.3 (24.2)	30.9 (24.1)	25.6 (24.1)	0.005
Median [Min, Max]	20.4 [0.0, 161.4]	23.6 [0.1, 113.6]	18.7 [0.0, 161.4]	
Missing	20 (3.6%)	14 (7.7%)	6 (1.6%)	
**MHT status**				
Never taken	259 (47.2%)	88 (48.1%)	171 (46.7%)	0.51
Taken before	213 (38.8%)	74 (40.4%)	139 (38.0%)	
Taking at sampling	70 (12.8%)	19 (10.4%)	51 (13.9%)	
Missing	7 (1.3%)	2 (1.1%)	5 (1.4%)	
**Statin status**				
Never taken	272 (49.5%)	86 (47.0%)	186 (50.8%)	0.76
Taken before	47 (8.6%)	15 (8.2%)	32 (8.7%)	
Taking at sampling	52 (9.5%)	19 (10.4%)	33 (9.0%)	
Missing	178 (32.4%)	63 (34.4%)	115 (31.4%)	
**Smoking** **(packs per year)**				
Mean (SD)	6.08 (9.57)	6.46 (9.73)	5.89 (9.50)	0.30
Median [Min, Max]	0.950 [0, 64.2]	1.65 [0, 49.3]	0.800 [0, 64.2]	
Missing	3 (0.5%)	3 (1.6%)	0 (0%)	
**Alcohol intake** **(g per week)**				
Mean (SD)	58.2 (69.9)	60.0 (70.9)	57.3 (69.5)	0.88
Median [Min, Max]	37.0 [0, 575]	37.0 [0, 292]	37.0 [0, 575]	
Missing	2 (0.4%)	2 (1.1%)	0 (0%)	
**Ever given birth**				
Never given birth	78 (14.2%)	27 (14.8%)	51 (13.9%)	0.80
Has given birth	470 (85.6%)	155 (84.7%)	315 (86.1%)	
Missing	1 (0.2%)	1 (0.5%)	0 (0%)	
**ER status**				
Negative	–	18 (9.8%)	–	
Positive	–	137 (74.9%)	–	
Missing	–	28 (15.3%)	–	
**PR status**				
Negative	–	44 (24.0%)	–	
Positive	–	109 (59.6%)	–	
Missing	–	30 (16.4%)	–	
**HER2 status**				
Negative	–	136 (74.3%)	–	
Positive	–	14 (7.7%)	–	
Missing	–	33 (18.0%)	–	
**Invasiveness**				
Invasive	–	99 (54.1%)	–	
Carcinoma in situ	–	19 (10.4%)	–	
Missing	–	65 (35.5%)	–	
**Tumor size**				
< 20 mm	–	43 (23.5%)	–	
>= 20 mm	–	17 (9.3%)	–	
Missing	–	123 (67.2%)	–	
**Lymph node metastasis**				
No	–	143 (78.1%)	–	
Yes	–	15 (8.2%)	–	
Missing	–	25 (13.7%)	–	
**Nottingham Histologic Grade**				
1	–	31 (16.9%)	–	
2	–	68 (37.2%)	–	
3	–	72 (39.3%)	–	
Missing	–	12 (6.6%)	–	

Abbreviations: Body mass index (BMI), Menopausal hormone therapy (MHT), Estrogen receptor (ER), Progesterone receptor (PR), Human epidermal growth factor receptor 2 (HER2).

### Identifying protein biomarkers of case-control status

A set of 54 proteins were associated with case-control status with a nominal *p* < 0.05 in at least one of the three conditional logistic regression models tested (data not shown). However, none remained significant after adjustment for multiple testing (FDR > 0.05).

### Unsupervised clustering of participants based on their protein profiles

Before clustering, we adjusted the proteomics data for a selected set of covariates. The impact of BMI, age of the women at sampling, and study entry date (as a proxy for sample age) on the protein data were analyzed by projecting the data to two dimensions using Uniform Manifold Approximation and Projection (UMAP) (**Supplementary Fig. S4**) and by associating protein levels with BMI, age and entry date in a combined linear model. The linear association resulted in significant (*p* < 0.05) associations for 305, 415, and 57 proteins for BMI, age, and entry date, respectively. Thus, when considering both the overall impact on the measured proteins and the effect on individual proteins, the age of the women had the strongest influence on the measured proteins, followed by BMI and with a limited effect of entry date. The experimental proteomics data were therefore adjusted for BMI, age of the women, and study entry date prior to further analyses. Five individuals lacked information on BMI and were therefore excluded, leaving 573 samples (181 cases, 363 controls, 29 doubles) for analysis. 552 unique antibodies with 552 unique targets were left after removing antibodies with the same target **(Additional file 1**)**.**

**Fig. 2:**
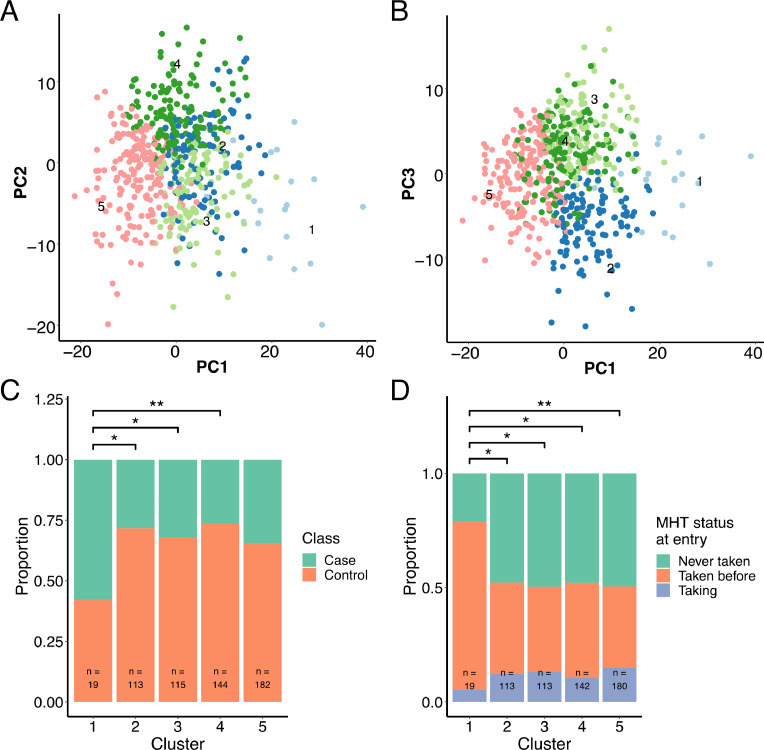
Principal component analysis (PCA) of each participant's protein profile plotted with **(A)** PC1/PC2 and **(B)** PC1/PC3. Each dot represents one participant that is colored by cluster. The stacked bar plots show comparisons between the five clusters in proportions: **(C)** Cases and controls, where doubles were treated as controls as they were all cancer-free at study entry; **(D)** Participants who were taking MHT prior to sample collection, at time of sampling, or never. Asterisks symbolize Fisher's exact test p-values (*: *p*<0.05, **: *p*<0.01) for pairwise comparisons between clusters.

To identify patterns in the proteomics data that grouped individuals into clusters, we performed archetypal analysis. We applied the Unit Invariant Knee method to identify the optimal number of clusters (as described in the supplementary material) (**Supplementary Fig. S5**) that would balance simplicity with adequate stratification of the data. This resulted in 5 clusters with 19, 113, 115, 144, and 182 participants, respectively (Fig. 2**A–D**), representing 3.3%, 19.7%, 20.0%, 25.1% and 31.8% of all tested subjects.

The mean Jaccard index (MJI) was calculated to assess cluster stability by repeating the clustering 150 times on bootstrap samples, randomly resampled with replacement. The MJIs between the most similar clusters for each clustering were summarized by taking the mean (**Supplementary methods, Unsupervised clustering**). The MJI for cluster 1–5 were 0.70 ± 0.29, 0.60 ± 0.12, 0.56 ± 0.16, 0.58 ± 0.14, and 0.61 ± 0.14 (**Supplementary Table S3**). To further assess the quality of the clustering, we determined the cluster membership of pairs of replicated samples and pairs of samples collected on different occasions from the same individual (double samples). We observed that replicate sample pairs significantly more often belonged to the same cluster than double sample pairs (**Supplementary Figs. S6, S7, Supplementary results**). This is in line with the difference in measured protein levels between replicate sample pairs being of purely technical origin. In contrast, differences in measured protein levels of the double pairs can be of both technical and biological origin due to the time elapsed between samplings. In addition, doubles pairs belonged more often to the same cluster than random pairs of samples. Thus, also showing that the protein profiles of the individual women did not substantially change between samplings. Taken together, this indicated that the clustering captures groups of individuals with similar protein profiles.

### Clinically characterizing the clusters of participants

Clusters of participants were defined at the protein level, and we proceeded to investigate how the stratification observed at the protein level might be reflected at the clinical level. We, therefore, contrasted a range of clinical variables across the clusters (Table 2 **and Supplementary Table S4**). Women belonging to cluster 1 had distinct clinical characteristics. Given that cluster 1 was the most stable cluster (MJI = 0.70) and the cluster with a unique protein profile, we focused the remaining analyses on this cluster. Cluster 1 consisted of women of a higher age than clusters 2 and 4 (*p* < 0.05, **Supplementary Fig. S8A, B**), despite the proteomics data being adjusted for age prior to archetype clustering. Consequently, 18 of the 19 women (95%) in cluster 1 were postmenopausal, while all other clusters included 71% to 77% of postmenopausal women. BMI and BMI-adjusted area-based breast density were not significantly different across clusters (**Supplementary Fig. S8**C**–D**). Cluster 1 had a mean and median dense area of 25.8 cm^2^ and 21.2 cm^2^, respectively (Table 2). Though the density for women in cluster 1 was not significantly different than the other clusters, it was substantially higher than a comparative sub-group of women of the same age. The group used for comparison were women within the same age range (63–65) and the same proportion of breast cancer cases from the KARMA cohort [35,36].

**Table 2:** Overview of the clinical characteristics of the archetype clusters.

	**1 (*N*** **=** **19)**	**2 (*N*** **=** **113)**	**3 (*N*** **=** **115)**	**4 (*N*** **=** **144)**	**5 (*N*** **=** **182)**
**Case control status**					
Case	11 (57.9%)	32 (28.3%)	37 (32.2%)	38 (26.4%)	63 (34.6%)
Control	8 (42.1%)	81 (71.7%)	78 (67.8%)	106 (73.6%)	119 (65.4%)
**Age**					
Mean (SD)	63.7 (6.95)	58.7 (9.29)	59.7 (9.63)	58.5 (9.97)	59.1 (9.33)
Median [Min, Max]	65.0 [46.0, 76.0]	61.0 [40.0, 78.0]	63.0 [39.0, 81.0]	61.5 [40.0, 78.0]	62.0 [39.0, 81.0]
**BMI**					
Mean (SD)	24.2 (4.10)	25.5 (3.96)	25.6 (3.70)	25.6 (4.65)	25.3 (4.24)
Median [Min, Max]	23.7 [17.9, 33.9]	24.8 [18.8, 37.0]	25.2 [18.5, 36.3]	24.8 [18.4, 44.2]	25.0 [17.6, 49.0]
**MHT status**					
Never taken	4 (21.1%)	54 (47.8%)	56 (48.7%)	68 (47.2%)	89 (48.9%)
Taken before	14 (73.7%)	45 (39.8%)	42 (36.5%)	59 (41.0%)	64 (35.2%)
Taking at entry	1 (5.3%)	14 (12.4%)	15 (13.0%)	15 (10.4%)	27 (14.8%)
Missing	0 (0%)	0 (0%)	2 (1.7%)	2 (1.4%)	2 (1.1%)
**Statin status**					
Never taken	12 (63.2%)	62 (54.9%)	55 (47.8%)	68 (47.2%)	93 (51.1%)
Taken before	1 (5.3%)	9 (8.0%)	8 (7.0%)	13 (9.0%)	17 (9.3%)
Taking at entry	2 (10.5%)	14 (12.4%)	9 (7.8%)	8 (5.6%)	19 (10.4%)
Missing	4 (21.1%)	28 (24.8%)	43 (37.4%)	55 (38.2%)	53 (29.1%)
**Menopausal status**					
Premenopausal	1 (5.3%)	33 (29.2%)	27 (23.5%)	41 (28.5%)	46 (25.3%)
Postmenopausal	18 (94.7%)	80 (70.8%)	88 (76.5%)	103 (71.5%)	136 (74.7%)
**Dense area (cm2)**					
Mean (SD)	25.8 (20.7)	29.0 (27.2)	28.6 (28.7)	30.0 (26.0)	25.6 (20.0)
Median [Min, Max]	21.2 [1.3, 73.7]	23.6 [0.0, 124.0]	19.8 [0.0, 161.0]	21.0 [0.0, 119.0]	20.4 [0.0, 86.9]
Missing	0 (0%)	10 (8.8%)	0 (0%)	3 (2.1%)	7 (3.8%)
**BMI- and age-adjusted dense area (cm2)**					
Mean (SD)	20.7 (18.3)	21.6 (24.7)	22.7 (26.6)	23.0 (22.0)	18.7 (18.4)
Median [Min, Max]	15.5 [−4.4, 70.3]	12.7 [−12.7, 109.0]	16.4 [−12.6, 161.0]	16.5 [−9.4, 90.2]	14.8 [−13.3, 76.1]
Missing	0 (0%)	10 (8.8%)	0 (0%)	3 (2.1%)	7 (3.8%)
**Smoking (packs per year)**					
Mean (SD)	7.34 (9.69)	7.08 (10.0)	6.24 (10.7)	5.68 (8.01)	5.21 (9.33)
Median [Min, Max]	1.50 [0, 29.1]	1.50 [0, 46.6]	0 [0, 49.3]	1.50 [0, 42.9]	0.450 [0, 64.2]
Missing	0 (0%)	1 (0.9%)	1 (0.9%)	0 (0%)	0 (0%)
**Alcohol intake** **(g per week)**					
Mean (SD)	70.4 (69.6)	49.2 (64.4)	52.4 (60.1)	76.9 (76.4)	51.3 (70.2)
Median [Min, Max]	37.0 [0, 261]	37.0 [0, 362]	37.0 [0, 273]	37.0 [0, 292]	37.0 [0, 575]
Missing	0 (0%)	0 (0%)	1 (0.9%)	0 (0%)	0 (0%)
**Ever given birth**					
Never given birth	5 (26.3%)	16 (14.2%)	17 (14.8%)	13 (9.0%)	30 (16.5%)
Has given birth	14 (73.7%)	97 (85.8%)	98 (85.2%)	131 (91.0%)	152 (83.5%)

Abbreviations: Body mass index (BMI), Menopausal hormone therapy (MHT).

**Fig. 3:**
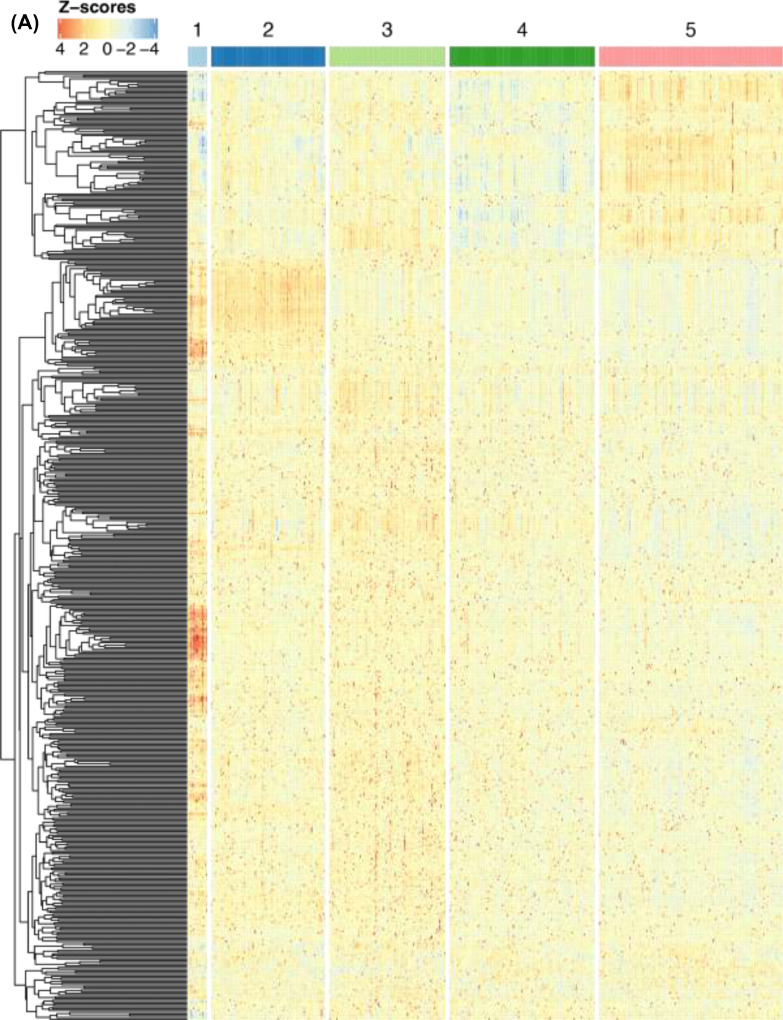

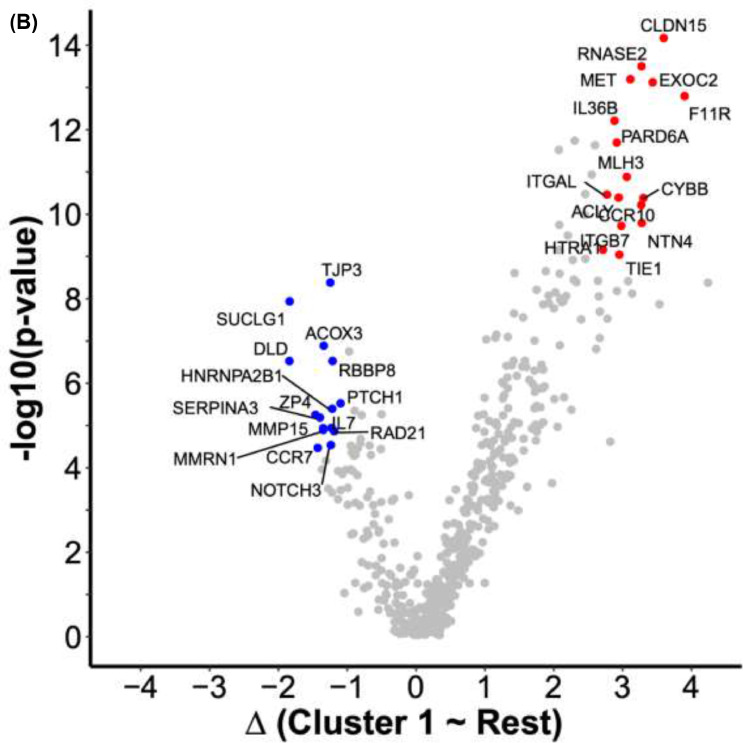
Proteomic characterization of clusters. **(A)** Heatmap of z-scores obtained from normalized, centered, and scaled MFI data. The dendrogram shows proteins (rows) in hierarchical clusters based on Euclidean distances. The participants (columns) are ordered into the archetype clusters they were assigned to. **(B)** Volcano plot of differentially abundant proteins in cluster 1 compared to samples in the remaining clusters. The x-axis represents the differences in median protein levels determined for each group using the normalized MFI values. Blue: A subset of 14 proteins with lower relative plasma levels were selected from the union of the 25 proteins with the lowest p-values and the 25 proteins with the largest decrease in abundance levels. Red: A subset of 16 proteins with higher relative plasma levels selected from the union of the 25 proteins with the lowest p-values and the 25 proteins with the largest increase in abundance levels.

There was a significantly greater proportion of breast cancer cases in cluster 1 compared to clusters 2, 3, and 4 (all *p* < 0.05, Fig. 3**A**). Cluster 1 also had a significantly greater proportion of women who had taken MHT compared to the other clusters (all *p* < 0.05, Fig. 3**B**). Additionally, the proportion of women who had previously taken MHT prior to study entry but were not taking MHT at the time of blood sampling, was also significantly higher in cluster 1 (all *p* < 0.05, Fig. 3**C**). We observed no significant difference between clusters regarding the time from last MHT to study entry (**Supplementary Fig. S8E**). Cluster 1 contained a higher proportion of cases who had taken MHT ever (100% of cases) compared to other clusters (approximately 50% of cases) (**Supplementary Fig. S8F**).

Given that 101 of the women were currently using or had previously been treated with statins and that statin use has previously been shown to affect the plasma proteome [37,38], we wanted to exclude this as a possible confounding factor. We observed no significant difference between clusters regarding statin usage, neither when delineating by statin type nor grouping all statins (**Supplementary Fig. S9**). Lastly, we compared PRSs across clusters and found no significant difference. Also, no significant differences were observed when comparing only cases in cluster 1 with cases in the remaining clusters. Additionally, when comparing the PRS of all cases to all controls, the PRS was slightly higher for cases. However, this difference was not statistically significant. This could be due to the small sample sizes. (**Supplementary Fig. S10**).

Given that several cases and controls had previous cancer diagnoses, we reran the clinical comparison of the clusters where these individuals were excluded to ascertain that such previous cancer and related treatment was not driving the differences observed. We did not observe any major changes resulting from excluding these individuals (data not shown). Applied to only the subset of postmenopausal women, the archetypal analysis again found a small cluster of 16 women. This cluster resembled cluster 1 in terms of higher frequencies of cases and previous MHT users (*p* < 0.05; data not shown). In fact, 16 of 18 postmenopausal women from the original cluster 1 (88% overlap) were grouped together again. This supports the utility of the chosen approach.

### Investigating the proteomic differences between clusters of participants

Differences in protein levels between the clusters were reflected in a heatmap (Fig. 3**A**). Distinct patterns reflecting the differences in protein levels can be observed for all clusters but are most apparent for cluster 1. The differential abundance analysis comparing the protein profiles of women in cluster 1 with all other individuals yielded 393 (72% of all) proteins with higher levels, of which 245 had an FDR < 0.05. In contrast, there were 159 (28% of all) proteins with lower levels, 73 of which had an FDR < 0.05. There were no significantly enriched pathways neither from the ORA over-representation analysis nor the GSEA gene set enrichment analysis. However, this investigation was likely biased by the already highly selective design to target only a particular set of proteins in the circulation.

To provide insights into the proteomic signatures of cluster 1, we shortlisted those proteins unifying the lowest p-values and largest relative abundance differences. Compared to the rest of the participants and choosing the union of the 25 most significant and 25 most differentially abundant proteins of cluster 1 (Fig. 3**B**), there were 16 more abundant (Table 3) and 15 less abundant proteins (Table 4). The levels of PTCH1 and ZP4 were significantly associated with adjusted breast density (nominal *p* < 0.05) and MHT status (nominal *p* < 0.05) when performing linear regression and logistic regression, respectively. CCR7, MMRN1, HNRNPA2B1, RBBP8, ACOX3, TJP3, and MMP15 were associated with adjusted breast density (nominal *p* < 0.05), but not MTH status (**Supplementary Fig. S11**). MFI levels of PTCH1 and ZP4 were lower in cases than in controls and significantly lower if MHT had been used (**Supplementary Fig. S12**).

**Table 3:** Proteins with lower plasma levels in cluster 1 compared to the other clusters.

Gene name	ENSG ID	FDR	FC
F11R	ENSG00000158769	1,93E-11	3,88
CLDN15	ENSG00000106404	4,08E-12	3,57
EXOC2	ENSG00000112685	1,14E-11	3,41
CYBB	ENSG00000165168	1,56E-09	3,28
NTN4	ENSG00000074527	5,14E-09	3,26
RNASE2	ENSG00000169385	9,52E-12	3,25
CCR10	ENSG00000184451	2,14E-09	3,25
MET	ENSG00000105976	1,14E-11	3,09
MLH3	ENSG00000119684	6,56E-10	3,04
ITGB7	ENSG00000139626	5,40E-09	2,96
TIE1	ENSG00000066056	2,19E-08	2,93
ACLY	ENSG00000131473	1,56E-09	2,92
PARD6A	ENSG00000102981	1,52E-10	2,89
IL36B	ENSG00000136696	6,17E-11	2,86
ITGAL	ENSG00000005844	1,48E-09	2,75
HTRA1	ENSG00000166033	1,78E-08	2,69

Abbreviations: False discovery rate corrected p-value (FDR); Median fold change (FC).

**Table 4:** Proteins with higher levels in cluster 1 compared to the other clusters.

Gene name	ENSG ID	FDR	FC
DLD	ENSG00000091140	2,54E-06	−1,86
SUCLG1	ENSG00000163541	1,49E-07	−1,86
ZP4	ENSG00000116996	3,18E-05	−1,48
CCR7	ENSG00000126353	1,39E-04	−1,45
SERPINA3	ENSG00000196136	3,55E-05	−1,42
MMP15	ENSG00000102996	5,64E-05	−1,37
MMRN1	ENSG00000138722	6,12E-05	−1,37
ACOX3	ENSG00000087008	1,20E-06	−1,36
TJP3	ENSG00000105289	6,99E-08	−1,27
NOTCH3	ENSG00000074181	1,23E-04	−1,26
IL7	ENSG00000104432	5,62E-05	−1,25
HNRNPA2B1	ENSG00000122566	2,39E-05	−1,24
RBBP8	ENSG00000101773	2,54E-06	−1,24
RAD21	ENSG00000164754	6,45E-05	−1,21
PTCH1	ENSG00000185920	1,88E-05	−1,12

Abbreviations: False discovery rate corrected p-value (FDR); Median fold change (FC).

## Discussion

Applying an unsupervised analysis approach on plasma proteomic data from women of the KARMA breast cancer risk cohort, we identified a subset of individuals enriched by previous MHT users and a greater proportion of breast cancers. The women in this cluster were also older, predominantly postmenopausal, and had a larger mammographic dense area relative to their age. Characterization of circulating proteins driving the cluster found lower levels of proteins involved in cell adhesion and immunoregulation, and higher levels of proteins associated with DNA integrity, cell fate, metabolism, and the female reproductive system. This supports their putative roles in the development of breast cancer or mediation of risk factors.

At first, we attempted to use a traditional biomarker discovery approach to compare cases and controls. This did, however, not reveal any proteomic profiles to be significantly different between the two groups. Even though studies have suggested blood-based biomarkers for diagnosis, our observations are in line with previous literature reporting few or no protein biomarkers for overall early detection of breast cancer [[2], [3], [4],[6], [7], [8], [9]]. Likely, this reflects the already early detection possible by mammographic screening, the complex etiology and heterogeneity of the disease, and that effects from a multi-organ system contribute to the granularity in the circulating plasma proteome. Dedicated efforts such as KARMA or other trial studies, such as INSTIGO [39], present important efforts to systematically explore the information accessible in the circulating proteome. Indeed, most previous attempts have identified putative subtype-specific markers with, at best, limited performance in replication and independent validation efforts. Herein, we did also not detect any significant subtype-specific profiles of circulating proteins deemed useful for early detection.

A data-driven archetypal analysis was then used as an unsupervised approach to identify proteomic-based clusters. These were then linked to phenotypic or genotypic traits. This enabled the identification of associations between clusters of women with similar plasma profiles and risk factors for breast cancer. By clustering the participants on their proteomics data, we found one stable cluster (MJI = 0.7). In contrast, the assigned members of the remaining clusters showed higher interchangeability when the data was perturbed (MJI ≤ 0.61). Cluster assignments with lower MJI scores should therefore be interpreted with greater caution [29]. Clearer definition criteria for these clusters could be achieved by applying stricter inclusion cut-offs where any unassigned participants are further pooled into “in-between” groups corresponding to individuals who do not reliably belong to single clusters. This possibility is also one of the strengths of archetype analysis over more traditional and static clustering methods. The non-binary cluster membership offers greater flexibility to reflect the extent of the diverse processes of human biology. However, such investigations go beyond the scope of this work. Consequently, we chose to focus on the clearest difference observed between women in the most stable cluster 1 (MJI = 0.7) and the remaining cohort.

In cluster 1, in which 79% of all women were previous MHT users, we also found an overrepresentation of breast cancers, with 58% being cases compared to 28–35% in the other clusters. This confirms previous knowledge that the use of MHT is associated with an increased 5-year risk of breast cancer among postmenopausal women [40]. Of note, all cases in cluster 1 had previously been treated with MHT, while this was only true for half of the cases in other clusters. The proteomic signature of cluster 1 was associated with MHT usage, however, this was not driven by the current use of MHT. This suggested that previous use of MHT left a mark in the circulating proteome of these women and that this could be detected even years after discontinuing the treatment. MHT has previously been shown to affect several proteins in serum using mass spectrometry [41,42]. These studies indicate treatments effects on other circulating proteins that have been noted as potential cancer biomarkers. The investigations were limited to a year of MHT use and did not include samples from subjects after treatment. Individuals in our cluster 1 also had a greater mammographic density relative to their age which is a known risk factor for breast cancer. Interestingly, MHT usage is known to be associated with higher mammographic density in postmenopausal women [43], [44], [45], [46], [47]. In previous proteomics studies, increased levels of the epidermal growth factor receptor were discussed as a risk predictor for future breast cancer diagnosis among women using MHT [48]. However, to our current knowledge, no longitudinal and population-scaled studies have been performed to investigate the potential long-term effects of MHT on density. Our results suggest that such studies may be warranted. It is therefore not clear if the increased relative density observed in cluster 1 is due to the previous MHT use or other factors. Interestingly, the use of statins was not seen as a major driver of the protein profiles despite its known effects on the plasma proteome [37,38]. This supported the observed effect of MHT being specific for this class of drugs. Additionally, no effect of genetic risk was observed in our data. However, this could be due to the low sample size.

Investigating the proteins driving cluster 1, we found lower levels of circulating proteins regulating DNA repair/integrity (RBBP8, RAD21) and cell fate/replication (NOTCH3, TJP3, HNRNPA2), which play a role in cancer development. Concordantly, RBBP8, TJP3, and HNRNPA2 were also significantly negatively associated with mammographic density. Individuals in cluster 1 had higher circulating levels of proteins that may be linked to mammographic breast density and the accompanying mechanical stiffness. This included the cell junction and adhesion molecules CLDN15, ITGB7, F11R, and its receptor ITGAL, which are potentially involved in sensing stiffness in the breast tissue and activating cellular downstream signaling pathways to maintain tissue homeostasis [49], [50], [51], [52], [53]. These proteins were positively associated with mammographic density, though the associations were not significant. Reassuringly, we replicated positive associations between mammographic density and F11R [21]. In fact, F11R has been widely described in cancer development and progression, and the expression of F11R correlates with poor breast cancer prognosis [54,55]. Our current findings validate our previous results and support our hypothesis that F11R plays a role in regulating mammographic density and breast tissue composition.

In addition to the mentioned candidates of cluster 1, we also found decreased levels of proteins related to the female tissues: ZP4 and PTCH1. Across clusters, the two proteins were decreased for cases compared to controls and in MHT treated compared to untreated women. Both proteins are expressed in female tissues, and we found both proteins to be negatively associated with mammographic density. Interestingly, these were the only two cluster-1-specific proteins that were also significantly associated with MHT use. We, therefore, hypothesize that MHT might negatively affect the expression in female tissues and thereby affect the plasma abundance of these proteins. ZP4 was selected for inclusion in this study due to its role in the extracellular matrix (SBA1). It is primarily expressed by the ovary, placenta, and other tissues [23,56]. ZP4 is part of the extracellular matrix surrounding oocytes, and it has been linked to the fertilization processes [57,58]. The protein PTCH1 was included in this study as it has previously been linked to cancer (SBA3). As a protein found on the cell surface and the Golgi apparatus, it functions as a tumor suppressor, and mutations of the *PTCH1* gene have been associated with poor prognosis and increased recurrence of breast cancer [59]. PTCH1 is expressed more widely than ZP4 but is among many tissues, expressed in female tissues, especially the cervix and endometrium [23,56]. The two proteins, ZP4 and PTCH1, could therefore potentially represent an unknown link between MHT usage, female tissues, and mammographic breast density all leading to increased risk of breast cancer.

Strengths of our study reside in the utilized exploratory affinity-based proteomic assay. It provides novel opportunities for high-throughput screening for circulating proteins associated to risk factors, indicative for disease development in selected phenotypes. The experimental design allows combining different protein assays into one multiplexed approach. The method is attractive due to its minimal consumption of sample volumes. The method provided us with relative protein quantities in plasma that allowed an in-depth comparative analysis across thousands of samples [60]. This complements initial biomarker discovery efforts using mass spectrometry to study the effects of MHT [61]. Even though previous efforts demonstrated the possibility to detect differentially abundant proteins in pre-cancer samples [48,61], capturing the inter-individual heterogeneity of the circulating proteome across many samples, as observed even in healthy subjects [10], has not been considered extensively. There is, however, now also a growing awareness about how a chosen method influences the type of information obtained from plasma analyses [62]. As the initial case-control analyses provided limited insights, we had enough datapoints per donor to proceed with a data-driven, thus hypothesis-generating strategy. Strengths also include the centralized and standardized collection of high-quality blood samples, which is also evident from the fact that we observed no systematic differences at the protein level between sampling centers. Women donated non-fasting blood samples during the mammography screening visit, thus blood was drawn at different dates and times of day. Even though this may carry the risk that metabolic effects influenced the plasma proteomes, it allowed us to assume that heterogeneous sampling timepoints can reduce a systematic influence of sampling in our study. Additionally, the centrally managed questionnaire data and mammograms obtained from all KARMA cohort participants prior to diagnosis, as well as the quantitative assessment of mammographic density by STRATUS [63] are strengths of this study.

Weaknesses in our study can be seen in the low number of breast cancer cases available from prospective population studies. An initial sampling of participants was based on a classical case-control design with two matched controls for each breast cancer case. Therefore, the cohort of women included in this study was enriched for breast cancer cases compared to the general population. However, this enrichment of cases increased the chances of observing effects related to risk factors and case-control status where much larger numbers of participants would otherwise have been needed. Furthermore, we used plasma to identify proteomic signatures associated with breast cancer risk factors and early detection. As previously discussed [21], it remains to be ascertained how well alterations in circulating protein concentrations can reflect the physiological activities and changes in protein expression of the breast tissue. However, as we have shown here, it seems that several systemic processes are contributing to the physiological changes occurring in breast cancer patients. Since the plasma provides a window into processes occurring in multiple tissues in one go, the identified epithelial and stromal cell-specific proteins likely appear in the blood due to leakage or shedding. An elevated turnaround of proteins in breast tissue can lead to detecting these targets in the circulation. Even when using the very well characterized hence comprehensive KARMA cohort, information on tumor characteristics and risk factors was missing for some participants: Data specific to MHT subtypes, dosage, and duration of the treatment, as well as some information on tumor characteristics was not available. Despite previous evidence that MHT subtypes and dosage increase the risk for breast cancer [64,65], the proportion of missing data made such an analysis of different MHT drugs across clusters unreasonable. Exposure data in KARMA is self-reported, which may result in measurement bias. However, exposure data, mammograms, and blood samples were collected at the same time at KARMA study entry, and it is not likely that the participants knew about their mammographic density at the time of answering the questionnaire. Besides, non-differential misclassification of exposures would dilute, not strengthen, the reported associations. Additionally, questionnaire data on drug usage was supplemented with data from the Swedish drug prescription registry. Given the expected heterogeneity of the molecular phenotypes, possible influence from other yet unknown factors, diet, or metabolic states at the time point of sampling, a lack in power may have further weakened the statistical significance of our findings. Our observations prompt further validation in independent, prospective cohorts with datasets of comparable design and depth.

## Conclusion

Our findings suggest that the use of MHT may leave long-lasting fingerprints in the circulating proteome. Effects of the treatment could be detected in the circulating proteome even years after discontinuation. These effects were especially apparent for proteins associated with mammographic density, breast tissue composition, tumor development and progression, and the female reproductive system. Like previous studies, we did not identify immediately applicable plasma protein biomarkers for an early detection of breast cancer. Instead, we identified circulating proteins associated with previous MHT use, connecting to a higher frequency of women with breast tumors, greater age, and relatively greater mammographic density. The findings obtained from profiling population samples provide novel biological insights into putative pathological processes associated with MHT usage and breast cancer risk. Collectively, this suggests that rather than looking for biomarkers secreted by a developing tumor for early breast cancer detection, proteomic characterization of plasma might currently be more successfully aimed at identifying biomarkers that modify or explain the effects of known risk factors. Unsupervised analysis approaches may aid in this endeavor by providing novel hypotheses. Our findings need to be further validated in plasma and cellular assays with breast or other female tissue. Still, they convey that further integration of health and treatment trajectories needs to be considered when judging molecular phenotypes of disease.

## CRediT authorship contribution statement

**Cecilia E. Thomas:** Conceptualization, Visualization, Methodology, Formal analysis, Writing – original draft, Writing – review & editing. **Leo Dahl:** Visualization, Methodology, Formal analysis, Writing – original draft, Writing – review & editing. **Sanna Byström:** Conceptualization, Data curation, Writing – original draft, Writing – review & editing. **Yan Chen:** Formal analysis, Writing – review & editing, Writing – original draft. **Mathias Uhlén:** Writing – review & editing, Writing – original draft. **Anders Mälarstig:** Writing – review & editing, Writing – original draft. **Kamila Czene:** Writing – review & editing, Writing – original draft. **Per Hall:** Supervision, Conceptualization, Writing – original draft, Writing – review & editing. **Jochen M. Schwenk:** Conceptualization, Visualization, Supervision, Writing – original draft, Writing – review & editing. **Marike Gabrielson:** Conceptualization, Visualization, Methodology, Formal analysis, Writing – review & editing, Writing – original draft.

## Declaration of Competing Interest

The authors declare the following financial interests/personal relationships which may be considered as potential competing interests:

MU is one of the founders of Atlas Antibodies AB, a company that sells Human Protein Atlas antibodies used in this study. JMS acknowledge a relationship with Atlas Antibodies AB. The other authors declare no conflict of interest.

## Appendix Supplementary materials

Image, application 1

Image, application 2
