# Supplementary material for: Circulating proteins reveal prior use of menopausal hormonal therapy and increased risk of breast cancer
Source: Transl Oncol. 2022 Jan 13;17:101339. doi: 10.1016/j.tranon.2022.101339 (PMC8760550; doi:10.1016/j.tranon.2022.101339)
Supplement: Supplementary file 1 [file mmc1.docx]

Supplementary

Supplementary materials and methods

**Genotyping**

Genotyping data was available for 229 participants (178 cases, 48 controls, 3 doubles) and was obtained using either a custom Illumina iSelect genotyping array chip, which included 200K single nucleotide polymorphisms (SNPs), or the Oncoarray chip, which included 500K SNPs. A weighted polygenic risk score for breast cancer was calculated for each genotyped participant using a recently published set of 313 SNPs that reached genome-wide significance [1].

**Unsupervised clustering**

To avoid over-representing some proteins, paired antibodies were removed so that there was only one antibody per target protein. For each target with multiple antibodies targeting it, the antibody with the highest correlation between replicate samples was kept. One sample was identified as an outlier using principal component analysis (PCA) and was removed. Hierarchical clustering was performed to verify that the outlier ended up in its cluster (**Supplementary Figure S13**). The normalized median fluorescence intensity (MFI) values are relative measures of protein abundance allowing comparison across different samples but not between different proteins. To emphasize relative variations rather than quantitative variations in the data, the remaining data was scaled and centered.

The optimal number of archetypes was identified using the “find_optimal_kappas” function. The optimal number is estimated by computing the sum of the squared estimate of errors (SSE) of the method for different numbers of archetypes. To find the number of archetypes where the benefit of adding more archetypes is low, an elbow point is identified, where the decrease in SSE diminishes. This was done using the Unit Invariant Knee (UIK) method [2].

The stability of the clustering was assessed by bootstrap creating 150 bootstraps of the data by random sampling with replacement. Clustering was then performed on each bootstrap sample, and the results were compared to the clustering on the original data. After excluding samples not drawn in the resampling, each original cluster was matched to the most similar bootstrap cluster in each bootstrap using the Jaccard index (JI) via the size of the intersection divided by the size of the union of two sets. The mean Jaccard indices (MJI) across all 150 matches for each original cluster were then used to summarize the stability of each cluster.

Supplementary results

**Proteomic analyses**

The quality of the antibody profiles was assessed using duplicate measurements of 96 samples labeled and processed independently during an experiment. Normalizing the data for technical variation using AbsPQN increased the median correlation between matching pairs of replicated samples from 0.993 to 0.994 and between matching pairs of doubles from 0.987 to 0.990 (**Supplementary Figure S14**). The multi-MA normalization made the mean MFIs of the 96-well plates on each 384-well plate more similar. Comparing Euclidean distance between 30 pairs of doubles and an equal number of pairs of random samples shows that the distance between doubles is smaller than the distance between random samples **(Supplementary figure S15A)**. Replicates also had a shorter Euclidean distance compared to random samples, when comparing 94 pairs of replicated samples to 94 pairs of random samples **(Supplementary figure S15B).**

During the antibody quality control, 15 unique antibodies were flagged for having a high MFI in empty wells, indicating unspecific binding. A set of 313 unique antibodies were flagged for having a low correlation between replicate samples. There were 25 unique antibodies being flagged for having a high correlation to anti-human IgG. Due to overlap in antibodies between the flagging criteria, 332 unique antibodies were flagged in total. This left 759 antibodies with 552 unique targets for the case-control analysis, after removal of flagged antibodies, control antibodies, and antibodies missing target information. For the remaining antibodies, we determined the coefficient of variation (CV) based on replicated measurements of pooled samples. The median of this technical CV (t.CV) was 6.1% **(Supplementary figure S15C)** and a median interquartile range (IQR) of 0.23 (based on normalized and log-transformed data, **Supplementary figure S15D**). Most variation in IQR is at the upper extreme, showing that most antibodies vary little compared to a few that stand for most of the biological variation. Correlations for antibodies in SBA1 were computed between the repeated assays, resulting in a median correlation of rho = 0.84 **(Supplementary figure S15E).**

**Judging cluster stability**

We used replicated samples and samples collected from the same individual at different points in time (denoted doubles) to judge the cluster membership assignment. Reassuringly, replicates and doubles were assigned more frequently to the same cluster than random pairs. In addition, the projection of the protein profiles using PCA or UMAP showed that both replicates and doubles resided near one another. This has also previously been observed in SBA-derived plasma data in a longitudinal analysis of healthy subjects [3]. This supports the observation that the groups cluster with samples with similar protein profiles. In the few cases where pairs of replicates and doubles were not assigned to the same distinct cluster, the membership of one or both members of the pair had been assigned almost equally to the two clusters.

Supplementary references

1. Mavaddat N, Michailidou K, Dennis J, Lush M, Fachal L, Lee A, Tyrer JP, Chen TH, Wang Q, Bolla MK *et al*: **Polygenic Risk Scores for Prediction of Breast Cancer and Breast Cancer Subtypes**. *Am J Hum Genet* 2019, **104**(1):21-34.

2. Christopoulos D: **Introducing Unit Invariant Knee (UIK) As an Objective Choice for Elbow Point in Multivariate Data Analysis Techniques**. *SSRN Electronic Journal* 2016.

3. Dodig-Crnkovic T, Hong MG, Thomas CE, Haussler RS, Bendes A, Dale M, Edfors F, Forsstrom B, Magnusson PKE, Schuppe-Koistinen I *et al*: **Facets of individual-specific health signatures determined from longitudinal plasma proteome profiling**. *EBioMedicine* 2020, **57**:102854.

Tables

***Table S1:*** *ATC codes for MHT and statins.*

| Drug | ATC code | Name | Type |
| --- | --- | --- | --- |
| Menopausal Hormone Therapy (MHT) | G03CA03 | estradiol | Estrogen only |
|  | G03CA04 | estriol |  |
|  | G03CA57 | conjugated estrogens |  |
|  | G03CX01 | tibolone |  |
|  | G03DC02 | norethisterone | Progestogen only |
|  | G03DA02 | medroxyprogesterone |  |
|  | G03DA04 | progesterone |  |
|  | G03FA01 | norethisterone and estrogen | Estrogen and progestogen combination |
|  | G03FA12 | medroxyprogesterone and oestrogen |  |
|  | G03FB05 | norethisterone and estrogen |  |
|  | G03FB06 | medroxyprogesterone and oestrogen |  |
|  | G03FB09 | levonorgestrel and estrogen |  |
| Statins | C10AA01 | simvastatin | Lipophilic |
|  | C10AA04 | fluvastatin |  |
|  | C10AA05 | Atorvastatin |  |
|  | C10AA08 | pitavastatin |  |
|  | C10AA03 | pravastatin | Hydrophilic |
|  | C10AA07 | rosuvastatin |  |

***Table S2****: Sample demographics including doubles.*

|  | Total (N=579) | Cases (N=183) | Controls (N=366) | Doubles (N=30) |
| --- | --- | --- | --- | --- |
| Age |  |  |  |  |
| Mean (SD) | 59.1 (9.51) | 59.6 (9.30) | 59.6 (9.28) | 49.3 (8.60) |
| Median  [Min, Max] | 62.0  [39.0, 81.0] | 62.0  [39.0, 81.0] | 62.0  [39.0, 81.0] | 46.5  [40.0, 72.0] |
| BMI |  |  |  |  |
| Mean (SD) | 25.4 (4.18) | 25.8 (3.78) | 25.5 (4.38) | 22.9 (3.16) |
| Median  [Min, Max] | 24.8  [17.6, 49.0] | 25.4  [18.5, 39.2] | 24.7  [17.6, 49.0] | 22.1  [18.8, 29.7] |
| Missing | 5 (0.9%) | 1 (0.5%) | 3 (0.8%) | 1 (3.3%) |
| Sampling center |  |  |  |  |
| Helsingborg Hospital | 313 (54.1%) | 95 (51.9%) | 188 (51.4%) | 30 (100%) |
| Landskrona Hospital | 23 (4.0%) | 7 (3.8%) | 16 (4.4%) | 0 (0%) |
| Skåne University Hospital, Lund | 20 (3.5%) | 7 (3.8%) | 13 (3.6%) | 0 (0%) |
| Stockholm South General Hospital | 223 (38.5%) | 74 (40.4%) | 149 (40.7%) | 0 (0%) |
| Menopausal status |  |  |  |  |
| Premenopausal | 150 (25.9%) | 45 (24.6%) | 85 (23.2%) | 20 (66.7%) |
| Postmenopausal | 428 (73.9%) | 137 (74.9%) | 281 (76.8%) | 10 (33.3%) |
| Missing | 1 (0.2%) | 1 (0.5%) | 0 (0%) | 0 (0%) |
| Dense area (cm2) |  |  |  |  |
| Mean (SD) | 28.1 (25.0) | 30.9 (24.1) | 25.6 (24.1) | 42.2 (33.3) |
| Median  [Min, Max] | 21.2  [0.001, 161] | 23.6  [0.060, 114] | 18.7  [0.001, 161] | 39.8  [0.665, 124] |
| Missing | 20 (3.5%) | 14 (7.7%) | 6 (1.6%) | 0 (0%) |
| MHT status |  |  |  |  |
| Never taken | 274 (47.3%) | 88 (48.1%) | 171 (46.7%) | 15 (50.0%) |
| Taken before | 226 (39.0%) | 74 (40.4%) | 139 (38.0%) | 13 (43.3%) |
| Taking at sampling | 72 (12.4%) | 19 (10.4%) | 51 (13.9%) | 2 (6.7%) |
| Missing | 7 (1.2%) | 2 (1.1%) | 5 (1.4%) | 0 (0%) |
| Statin status |  |  |  |  |
| Never taken | 292 (50.4%) | 86 (47.0%) | 186 (50.8%) | 20 (66.7%) |
| Taken before | 48 (8.3%) | 15 (8.2%) | 32 (8.7%) | 1 (3.3%) |
| Taking at sampling | 52 (9.0%) | 19 (10.4%) | 33 (9.0%) | 0 (0%) |
| Missing | 187 (32.3%) | 63 (34.4%) | 115 (31.4%) | 9 (30.0%) |
| Smoking (packs per year) |  |  |  |  |
| Mean (SD) | 5.96 (9.43) | 6.46 (9.73) | 5.89 (9.50) | 3.74 (5.88) |
| Median  [Min, Max] | 0.900  [0, 64.2] | 1.65  [0, 49.3] | 0.800  [0, 64.2] | 0  [0, 22.9] |
| Missing | 3 (0.5%) | 3 (1.6%) | 0 (0%) | 0 (0%) |
| Alcohol intake (g per week) |  |  |  |  |
| Mean (SD) | 58.0 (69.3) | 60.0 (70.9) | 57.3 (69.5) | 54.4 (58.5) |
| Median  [Min, Max] | 37.0  [0, 575] | 37.0  [0, 292] | 37.0  [0, 575] | 37.0  [0, 180] |
| Missing | 2 (0.3%) | 2 (1.1%) | 0 (0%) | 0 (0%) |
| Ever given birth |  |  |  |  |
| Never given birth | 83 (14.3%) | 27 (14.8%) | 51 (13.9%) | 5 (16.7%) |
| Has given birth | 495 (85.5%) | 155 (84.7%) | 315 (86.1%) | 25 (83.3%) |
| Missing | 1 (0.2%) | 1 (0.5%) | 0 (0%) | 0 (0%) |
| ER status |  |  |  |  |
| Negative | - | 18 (9.8%) | - | - |
| Positive | - | 137 (74.9%) | - | - |
| Missing | - | 28 (15.3%) | - | - |
| PR status |  |  |  |  |
| Negative | - | 44 (24.0%) | - | - |
| Positive | - | 109 (59.6%) | - | - |
| Missing | - | 30 (16.4%) | - | - |
| HER2 status |  |  |  |  |
| Negative | - | 136 (74.3%) | - | - |
| Positive | - | 14 (7.7%) | - | - |
| Missing | - | 33 (18.0%) | - | - |
| Invasiveness |  |  |  |  |
| Invasive | - | 99 (54.1%) | - | - |
| Carcinoma in situ | - | 19 (10.4%) | - | - |
| Missing | - | 65 (35.5%) | - | - |
| Tumor size |  |  |  |  |
| < 20 mm | - | 43 (23.5%) | - | - |
| >= 20 mm | - | 17 (9.3%) | - | - |
| Missing | - | 123 (67.2%) | - | - |
| Lymph node metastasis |  |  |  |  |
| No | - | 143 (78.1%) | - | - |
| Yes | - | 15 (8.2%) | - | - |
| Missing | - | 25 (13.7%) | - | - |
| Nottingham Histologic Grade |  |  |  |  |
| 1 | - | 31 (16.9%) | - | - |
| 2 | - | 68 (37.2%) | - | - |
| 3 | - | 72 (39.3%) | - | - |
| Missing | - | 12 (6.6%) | - | - |

***Table S3****: Bootstrapping analysis of cluster stability reported as MJI and SD for each cluster.*

| Cluster | 1 | 2 | 3 | 4 | 5 |
| --- | --- | --- | --- | --- | --- |
| MJI  (SD) | 0.702  (0.285) | 0.601  (0.123) | 0.555  (0.159) | 0.578  (0.136) | 0.605  (0.139) |

***Table 4****: Distribution of tumors characteristics for cases in archetype clusters.*

|  | **1 (N=11)** | **2 (N=32)** | **3 (N=37)** | **4 (N=38)** | **5 (N=63)** |
| --- | --- | --- | --- | --- | --- |
| **ER status** |  |  |  |  |  |
| Negative | 1 (9.1%) | 1 (3.1%) | 5 (13.5%) | 4 (10.5%) | 6 (9.5%) |
| Positive | 8 (72.7%) | 24 (75.0%) | 27 (73.0%) | 30 (78.9%) | 48 (76.2%) |
| Missing | 2 (18.2%) | 7 (21.9%) | 5 (13.5%) | 4 (10.5%) | 9 (14.3%) |
| **PR status** |  |  |  |  |  |
| Negative | 2 (18.2%) | 4 (12.5%) | 11 (29.7%) | 9 (23.7%) | 17 (27.0%) |
| Positive | 7 (63.6%) | 21 (65.6%) | 19 (51.4%) | 25 (65.8%) | 37 (58.7%) |
| Missing | 2 (18.2%) | 7 (21.9%) | 7 (18.9%) | 4 (10.5%) | 9 (14.3%) |
| **HER2 status** |  |  |  |  |  |
| Negative | 8 (72.7%) | 22 (68.8%) | 27 (73.0%) | 30 (78.9%) | 48 (76.2%) |
| Positive | 1 (9.1%) | 2 (6.2%) | 6 (16.2%) | 1 (2.6%) | 4 (6.3%) |
| Missing | 2 (18.2%) | 8 (25.0%) | 4 (10.8%) | 7 (18.4%) | 11 (17.5%) |
| **Invasiveness** |  |  |  |  |  |
| Cancer in situ | 2 (18.2%) | 6 (18.8%) | 3 (8.1%) | 6 (15.8%) | 10 (15.9%) |
| Invasive | 8 (72.7%) | 25 (78.1%) | 32 (86.5%) | 31 (81.6%) | 52 (82.5%) |
| Missing | 1 (9.1%) | 1 (3.1%) | 2 (5.4%) | 1 (2.6%) | 1 (1.6%) |
| **Tumor size**  **(TNM T)** |  |  |  |  |  |
| < 20mm | 4 (36.4%) | 18 (56.2%) | 20 (54.1%) | 16 (42.1%) | 36 (57.1%) |
| >= 20mm | 7 (63.6%) | 14 (43.8%) | 16 (43.2%) | 22 (57.9%) | 27 (42.9%) |
| Missing | 0 (0%) | 0 (0%) | 1 (2.7%) | 0 (0%) | 0 (0%) |
| **Lymph node metastasis**  **(TNM N)** |  |  |  |  |  |
| No | 10 (90.9%) | 32 (100%) | 34 (91.9%) | 31 (81.6%) | 59 (93.7%) |
| Yes | 1 (9.1%) | 0 (0%) | 2 (5.4%) | 6 (15.8%) | 4 (6.3%) |
| Missing | 0 (0%) | 0 (0%) | 1 (2.7%) | 1 (2.6%) | 0 (0%) |
| **Nottingham Histologic Grade** |  |  |  |  |  |
| 1 | 1 (9.1%) | 7 (21.9%) | 6 (16.2%) | 7 (18.4%) | 10 (15.9%) |
| 2 | 4 (36.4%) | 13 (40.6%) | 14 (37.8%) | 13 (34.2%) | 23 (36.5%) |
| 3 | 5 (45.5%) | 9 (28.1%) | 13 (35.1%) | 17 (44.7%) | 27 (42.9%) |
| Missing | 1 (9.1%) | 3 (9.4%) | 4 (10.8%) | 1 (2.6%) | 3 (4.8%) |

Abbreviations: Estrogen receptor (ER), Progesterone receptor (PR), Human epidermal growth factor receptor 2 (HER2), TNM classification of malignant tumors (TNM).

Figures


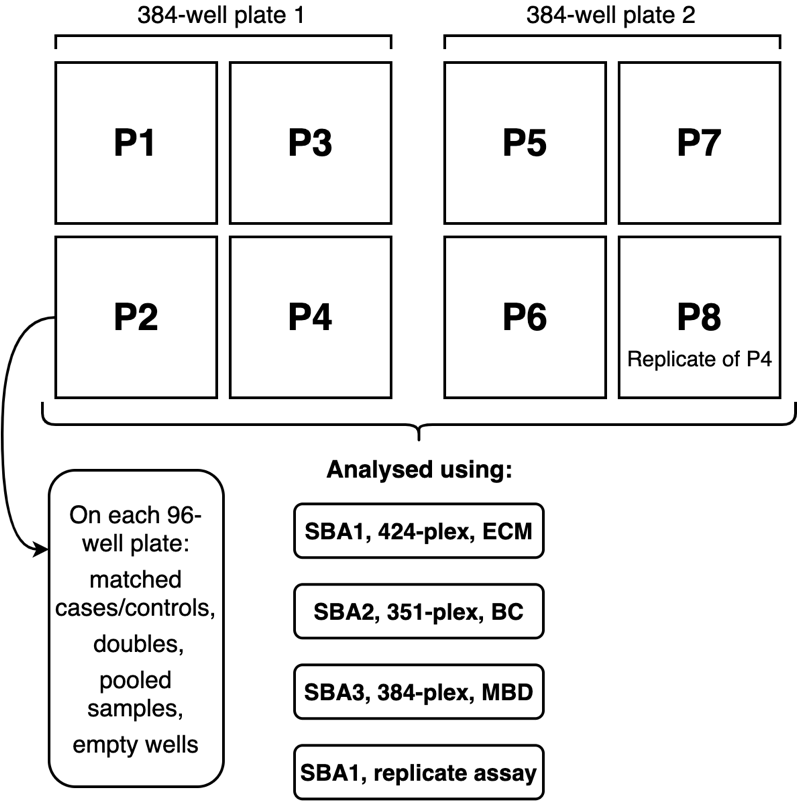


***Figure S1****: Sample layout.* *Eight 96-well plates of samples were assayed using three different antibody arrays, one of which was used in a replicate assay.*


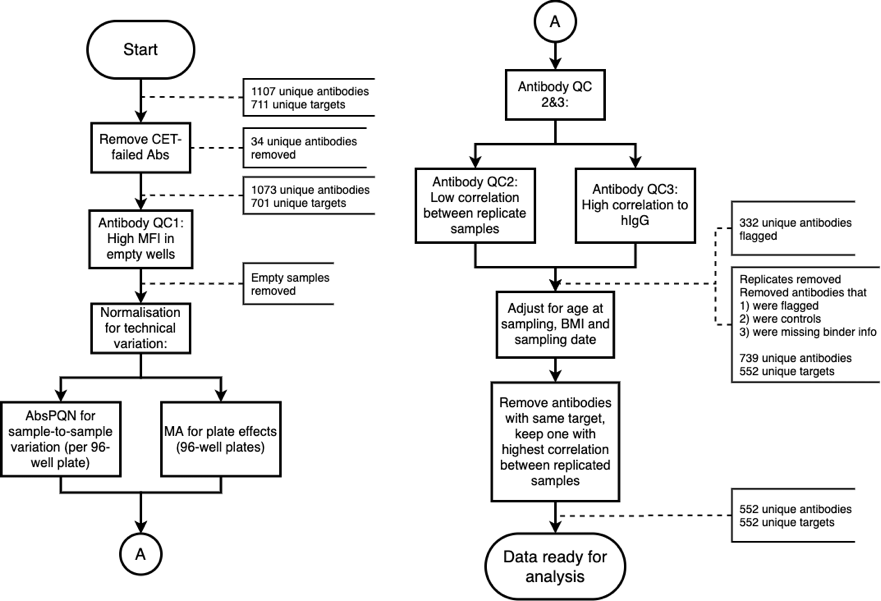


***Figure S2****: Data processing. After removal of antibodies that failed coupling, antibodies were filtered based on data quality. The data was normalized using AbsPQN for sample-to-sample variation and MA-normalization for plate effects. The data was adjusted for the confounding factors age, BMI, and study entry date. Lastly, antibodies targeting the same protein were filtered based on correlation to have one antibody per target.*

***
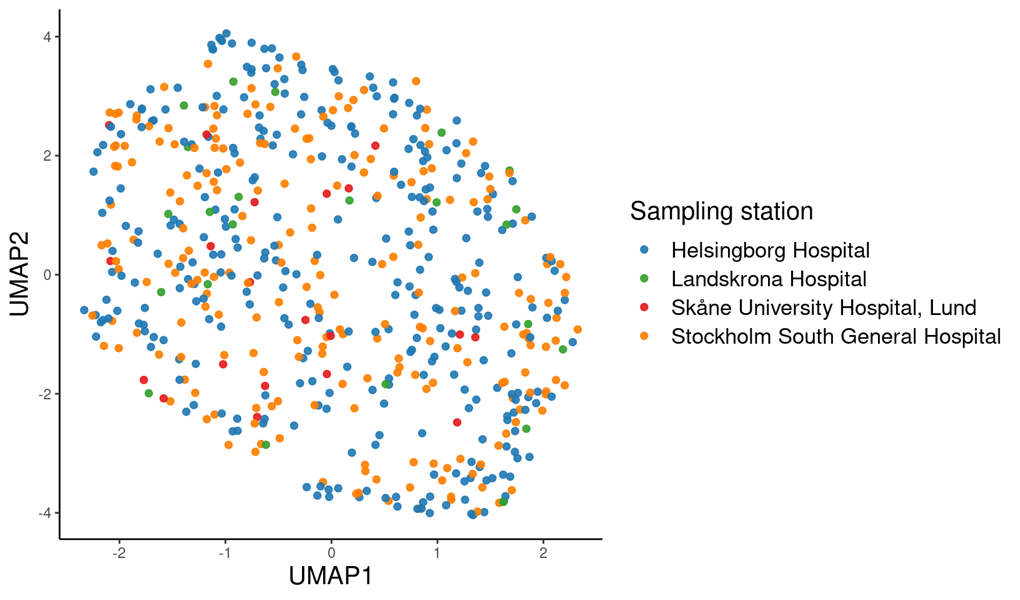
***

***Figure S2****:* *Dimensionality reduction plot (UMAP) of the participants based on their protein profiles colored by the sampling center.*


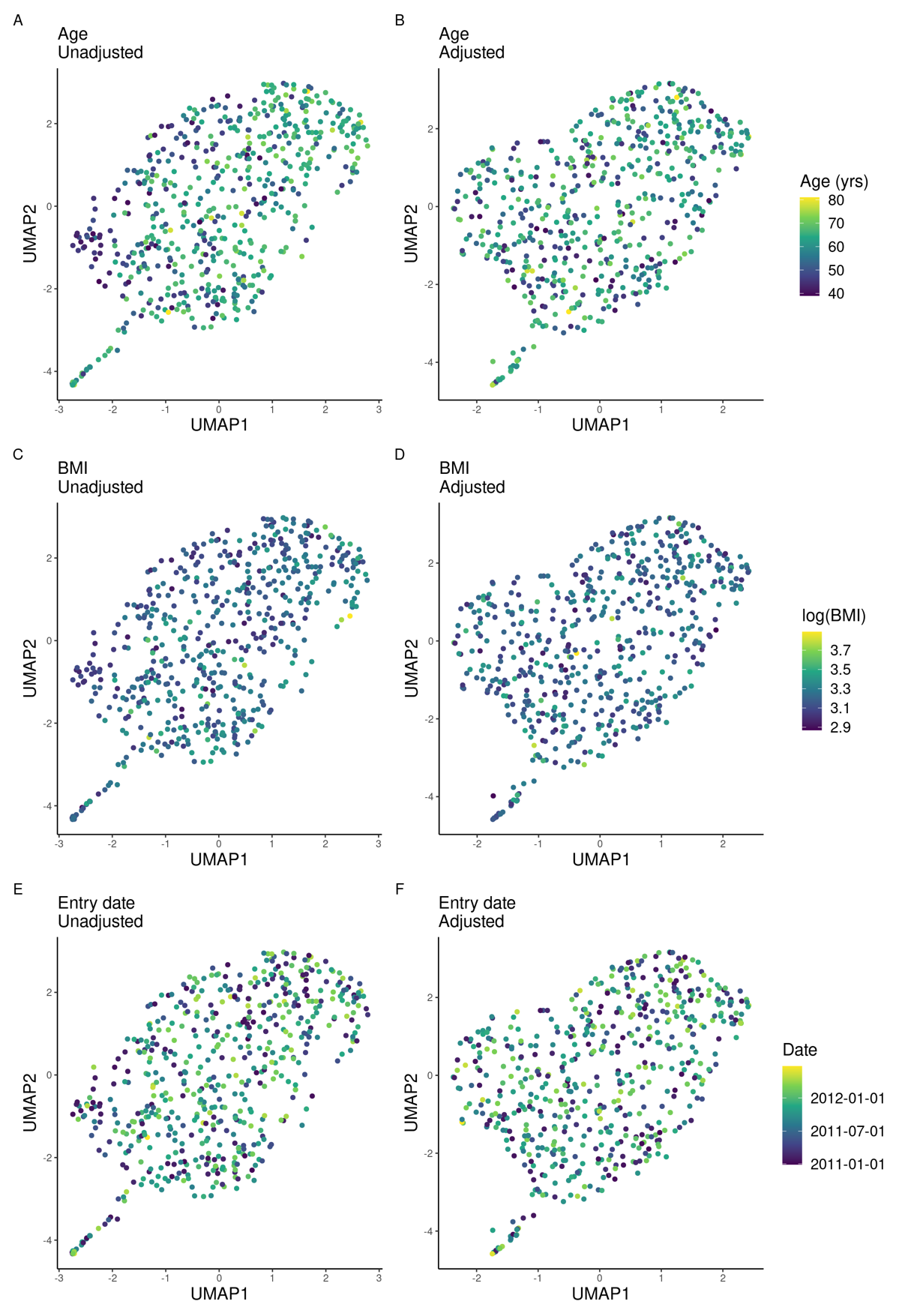


***Figure S4****: Dimensionality reduction plots (UMAP) of unadjusted data (left) and adjusted data (right), colored by the potential covariates age (A-B), BMI (C-D), and sampling date (E-F).*


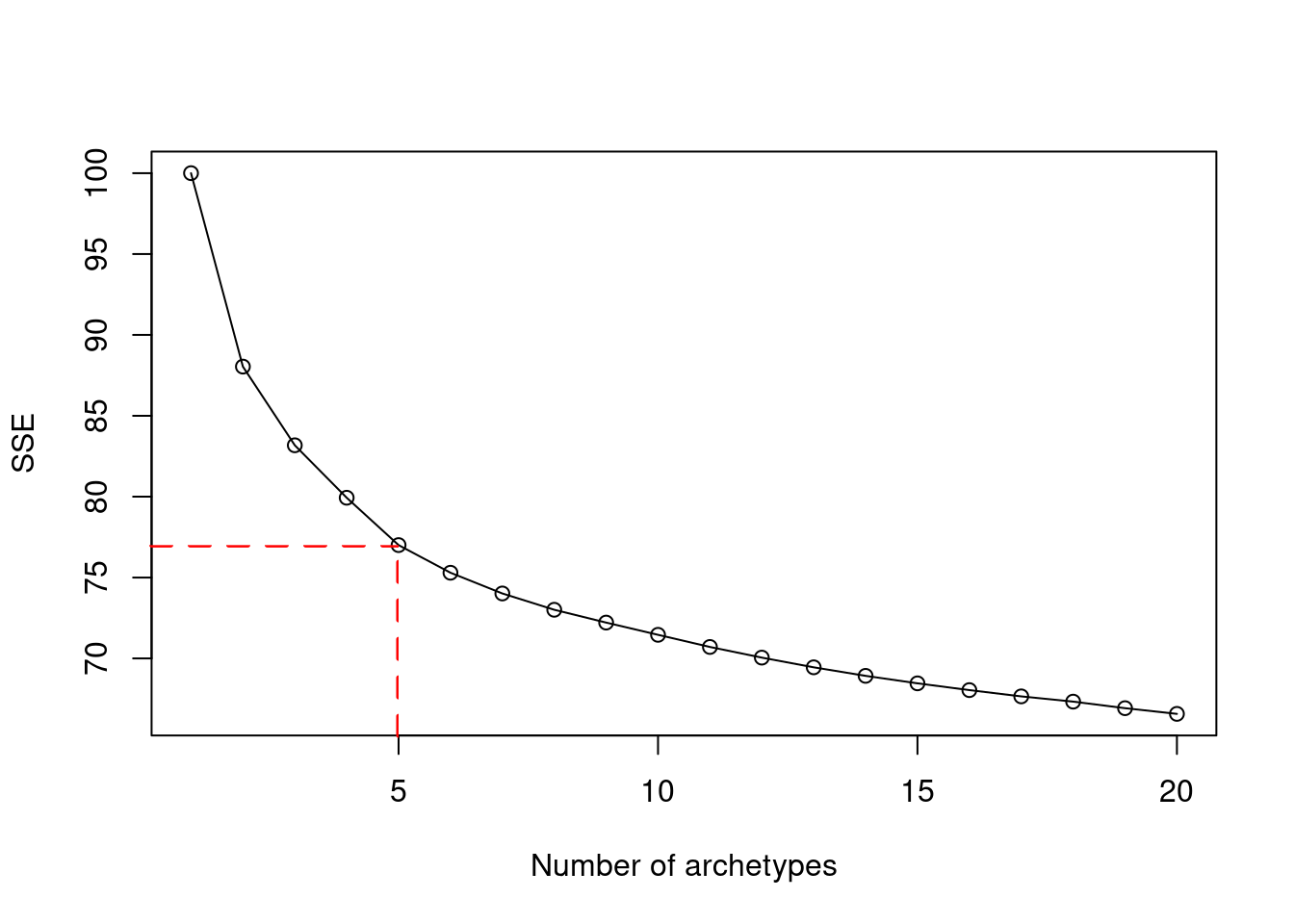


***Figure S5****: Identification of optimal number of archetypes. Scree plot of the sum of squared estimate of errors (SSE) of the archetypes plotted against the number of archetypes. The red lines mark the chosen number of archetypes (5).*


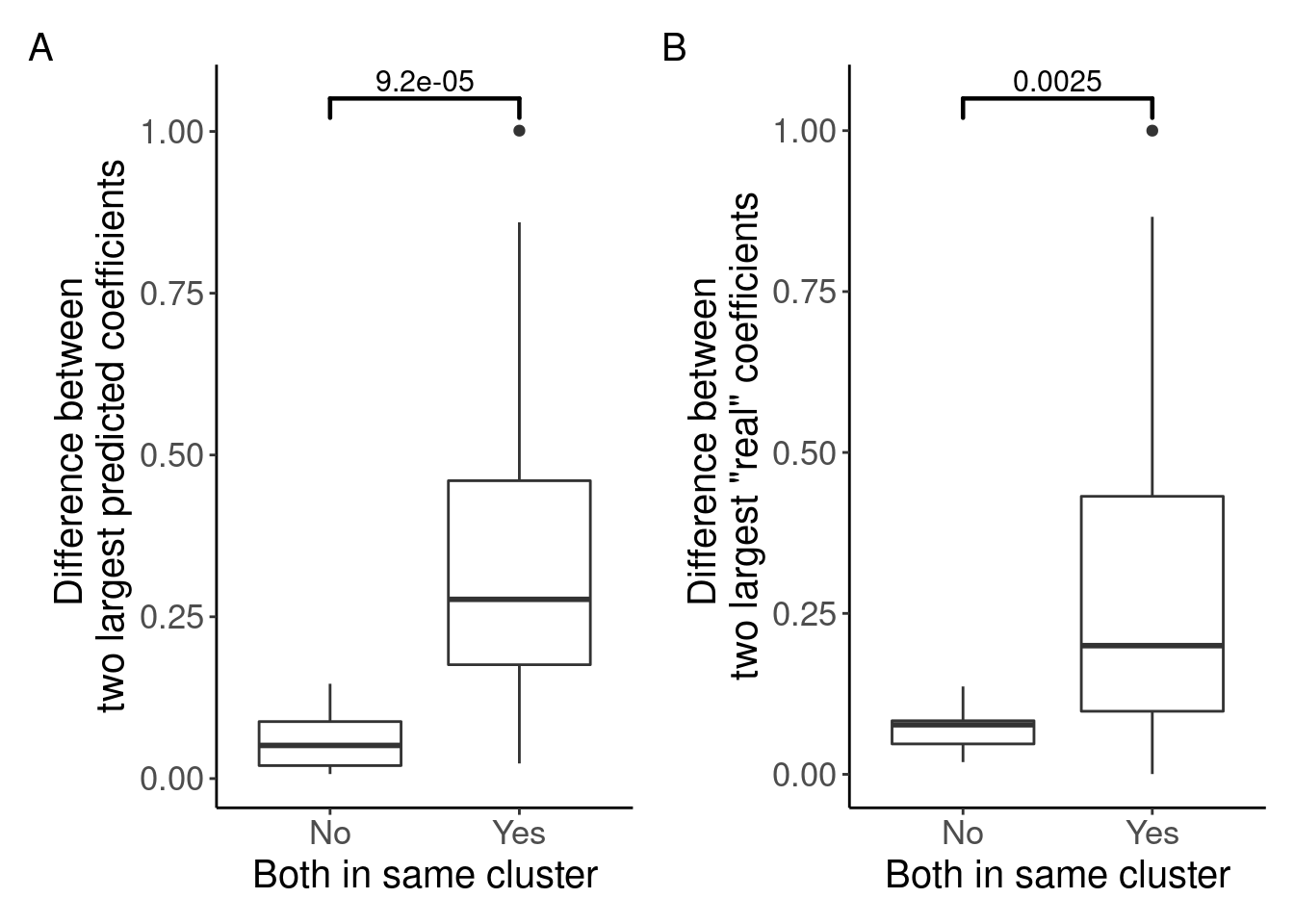

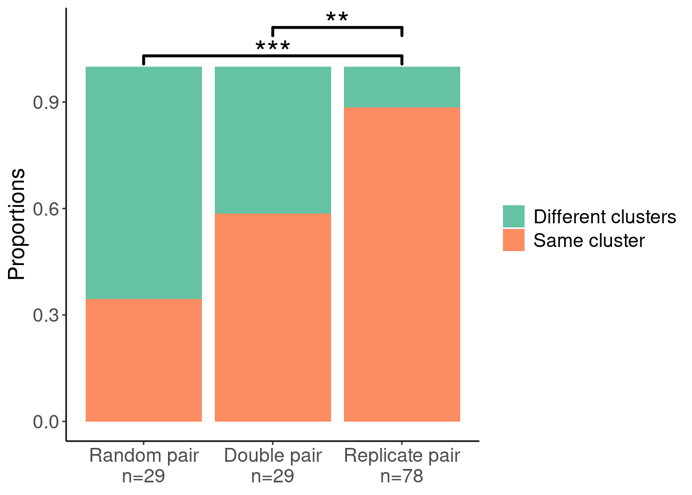


***Figure S6****:* ***(A-B)*** *Difference between largest and second-largest archetype coefficients for replicates that were in the same cluster and replicates that were in different clusters for* ***(A)*** *predicted coefficients and* ***(B)*** *the coefficients from the actual archetypal analysis. In* ***(C)****, the numbers of pairs of samples ending up in the same cluster or in different clusters for pairs of random samples, pairs of “doubles”, and pairs of replicate samples.* *Replicates that did not fall in the same cluster were generally at the edges of two clusters, thus their archetype coefficients (which determine the cluster) for the two clusters were almost identical. As shown in the PCA projections, these replicates were indeed positioned in close proximity to another, hence often close to the borders of the clusters. In comparison, replicates ending up in the same cluster had a larger difference between archetype coefficients – and more distant from the cluster borders –and therefore more reliably belonged to a single cluster (****Supplementary Figures S10 and S11****).*


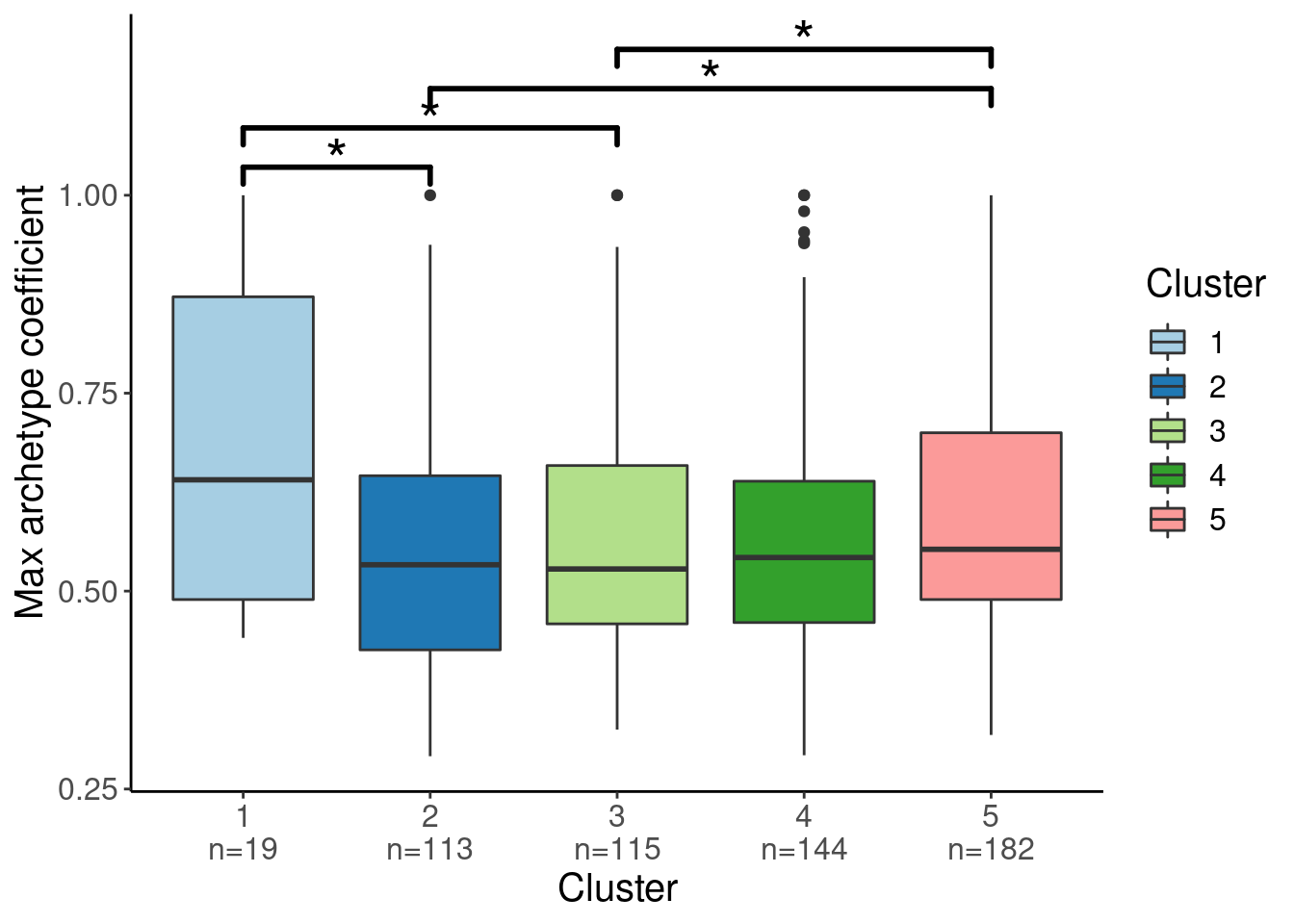


***Figure S7****: Largest archetype coefficients for samples in the different clusters.*

******

***Figure S8****: (A) Number of participants in each cluster. Distributions of (B) age, (C) BMI, and (D) mammographic density (cm2) (age- and BMI-adjusted) in the clusters. (E) Time in years between last use of MHT and study entry for the five clusters. (F) Numbers of participants in each cluster who have taken or not taken MHT, divided by case-control status. An asterisk symbolizes a Wilcoxon rank-sum test p < 0.05 by pairwise comparison.*


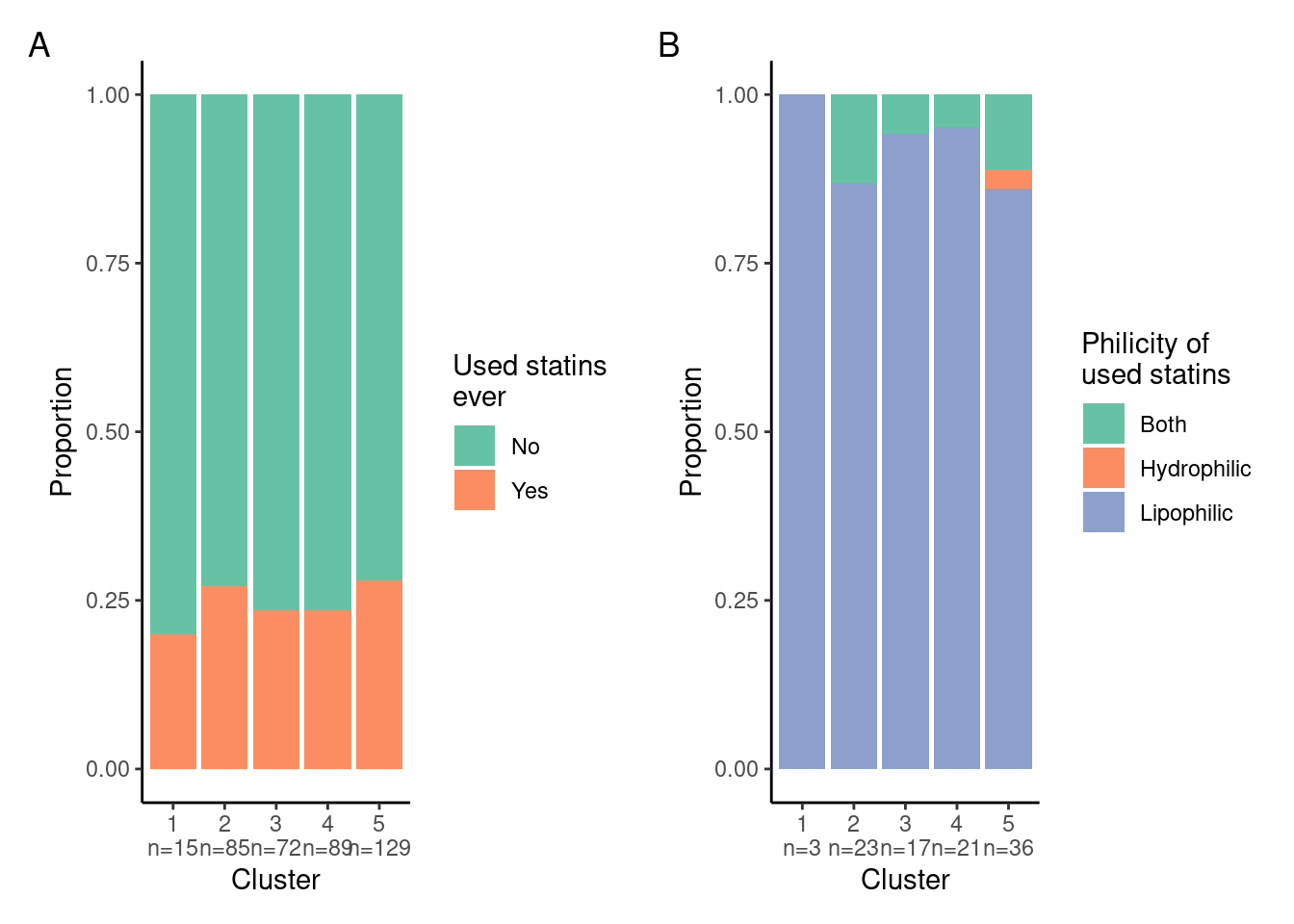


***Figure S9****: Proportions of participants who (A) used statins ever, and (B) the philicities of the used statins in the five clusters.*


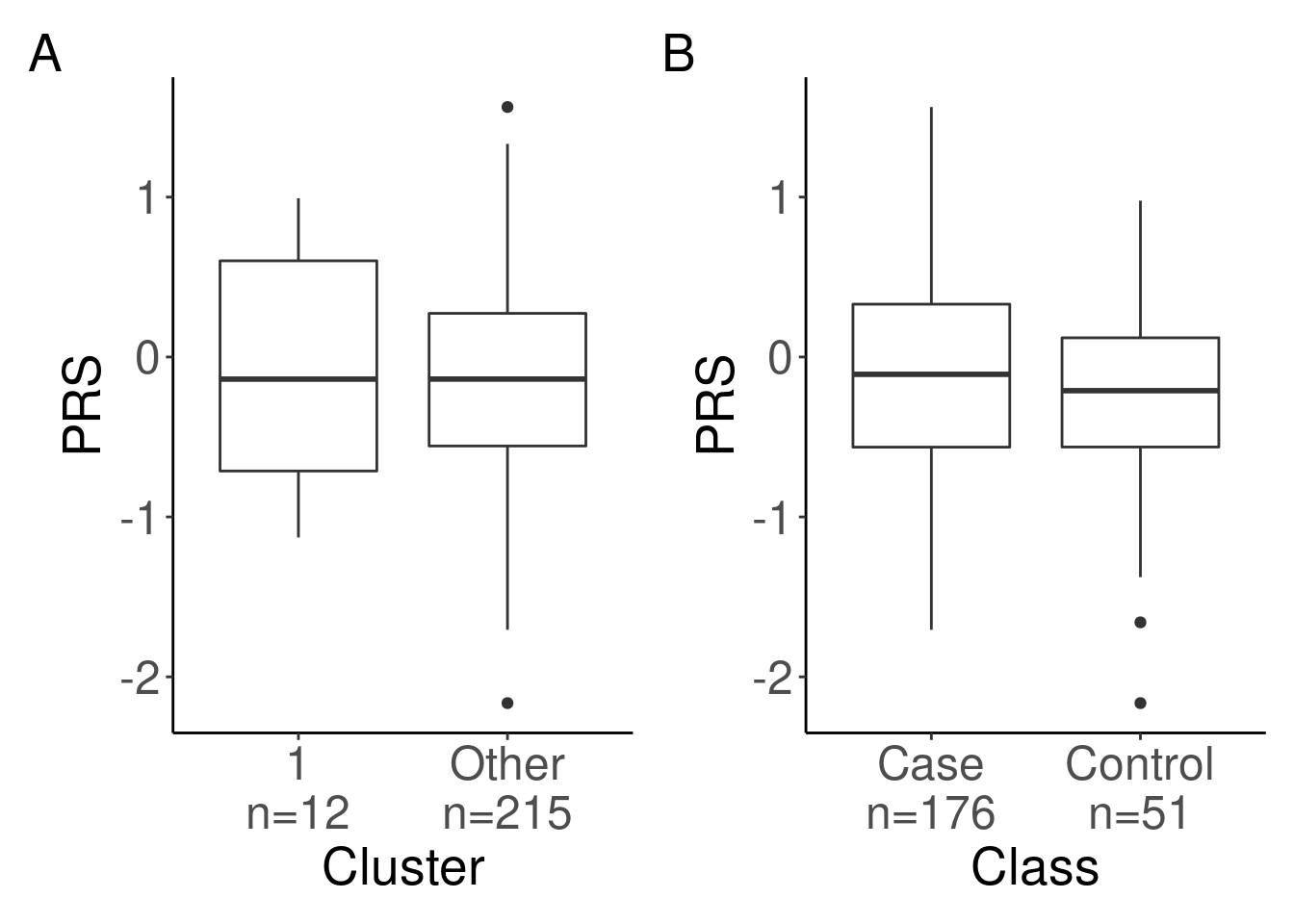


***Figure S10****: Distribution of polygenic risk score (PRS) across (A) cluster 1 and all other participants and (B) across all available cases and controls. Only one control was genotyped in cluster 1.*

***Figure S11****: Regression coefficients from linear and logistic regression between the values of the selected proteins of interest for cluster 1 and the adjusted dense area and (linear regression) and MHT status (logistic regression). Cells are colored by the regression coefficient and labeled with an asterisk representing the nominal p-value from the regression (*: p<0.05, **: p<0.01, ***: p<0.001). The direction of differential expression in cluster 1 is shown in the color bar at the top.*


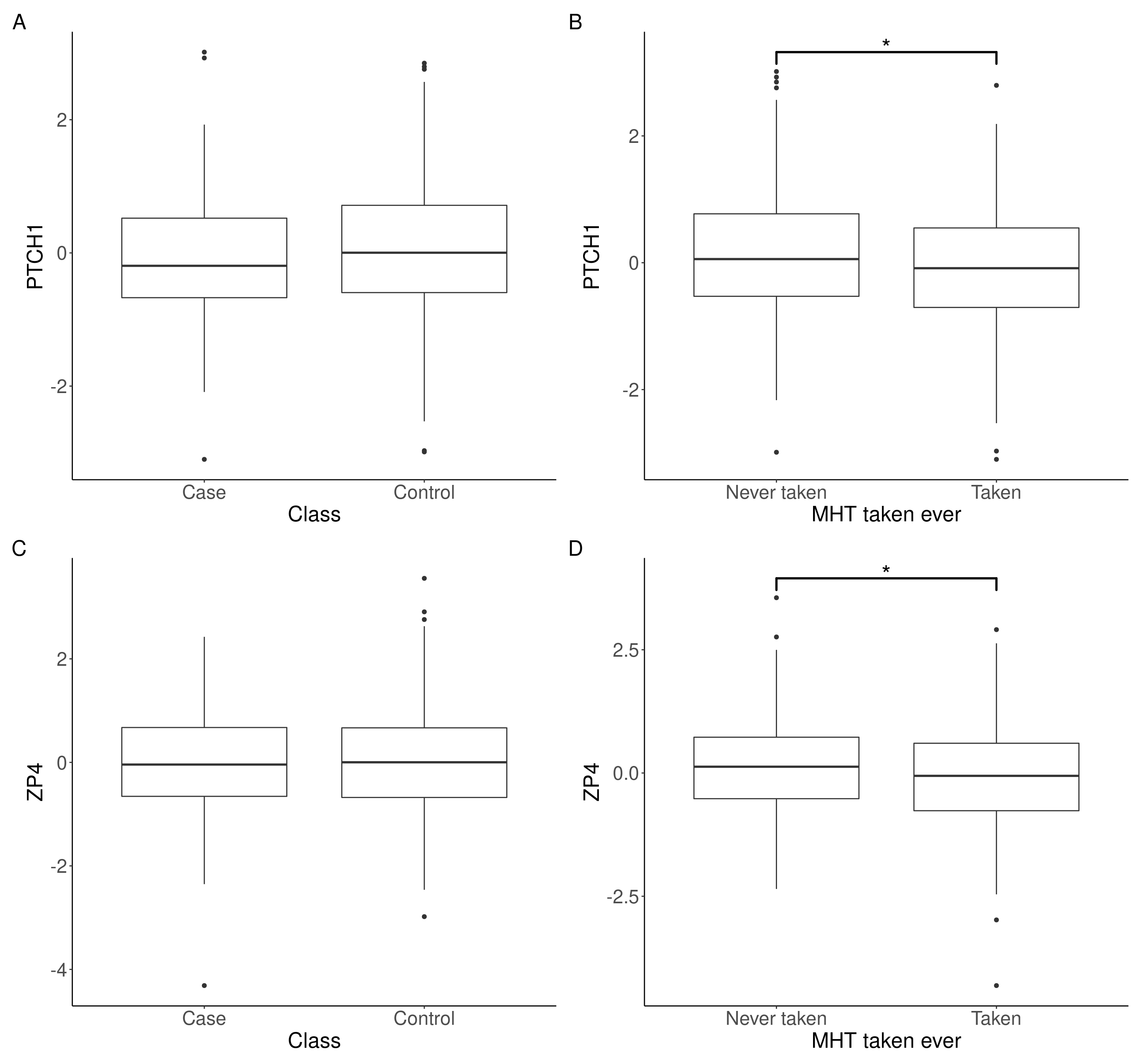


***Figure S12****: Distribution of MFI values (log-transformed, normalized, adjusted, scaled, and centered) for PTCH1 across (A) cases and controls and (B) MHT usage (0: never taken, 1: taken), and ZP4 across (C) cases and controls and (D) MHT usage.*


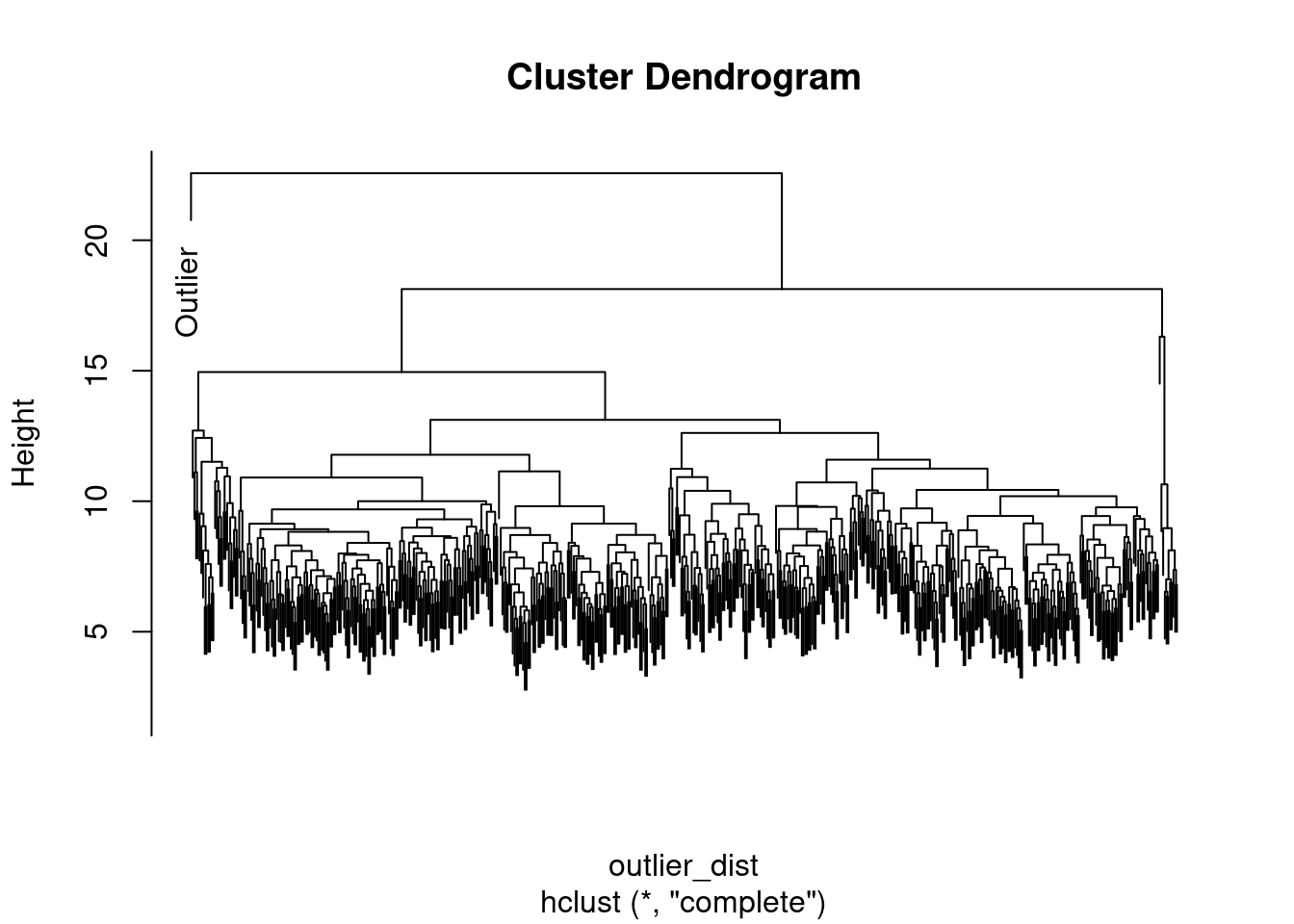


***Figure S13****: Hierarchical clustering to verify outlier sample.*


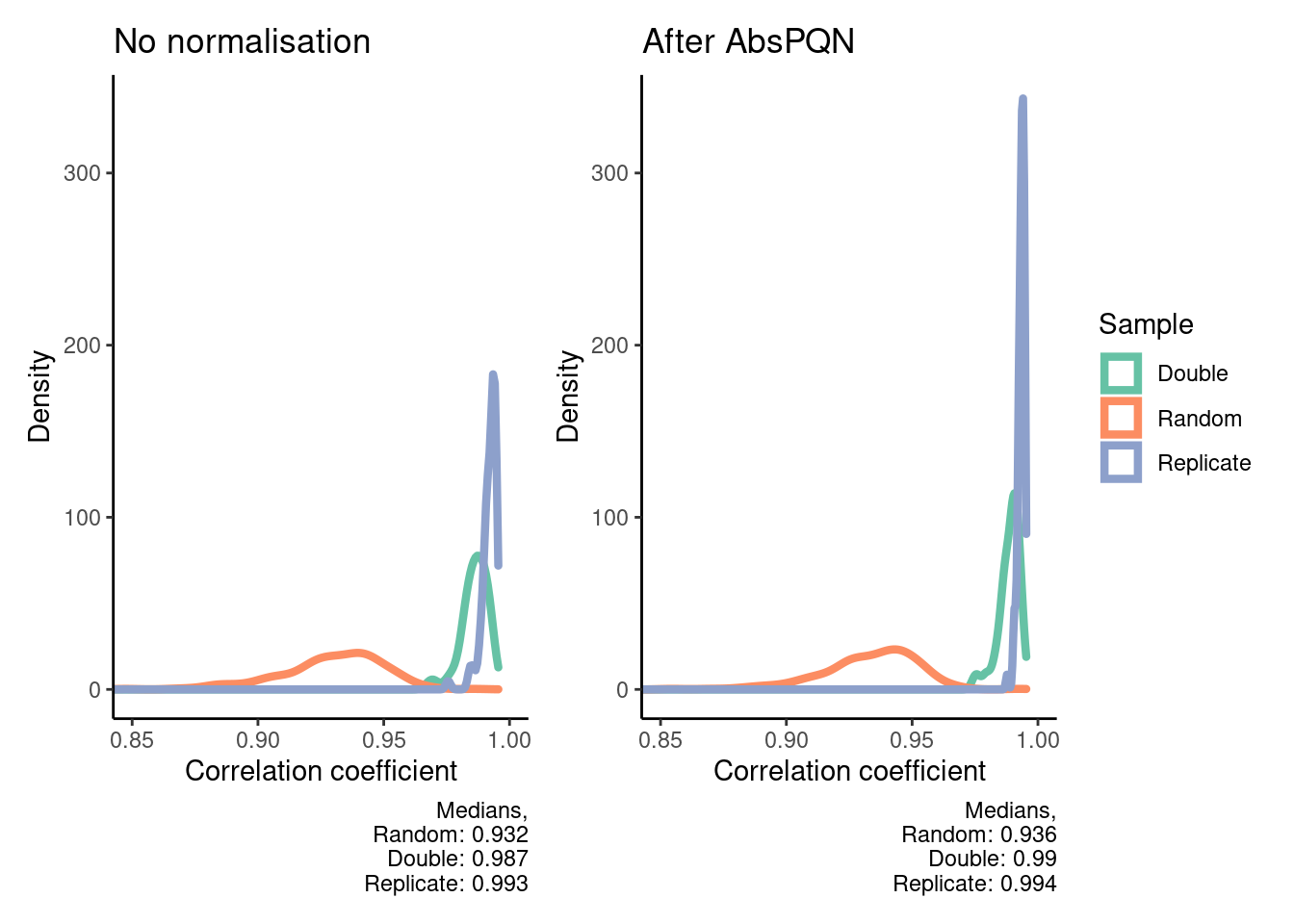


***Figure S14****: Effect of AbsPQN normalization on protein data. Densities of correlations between pairs of replicated samples, pairs of doubles, and pairs of random samples, before (left) and after (right) AbsPQN.*


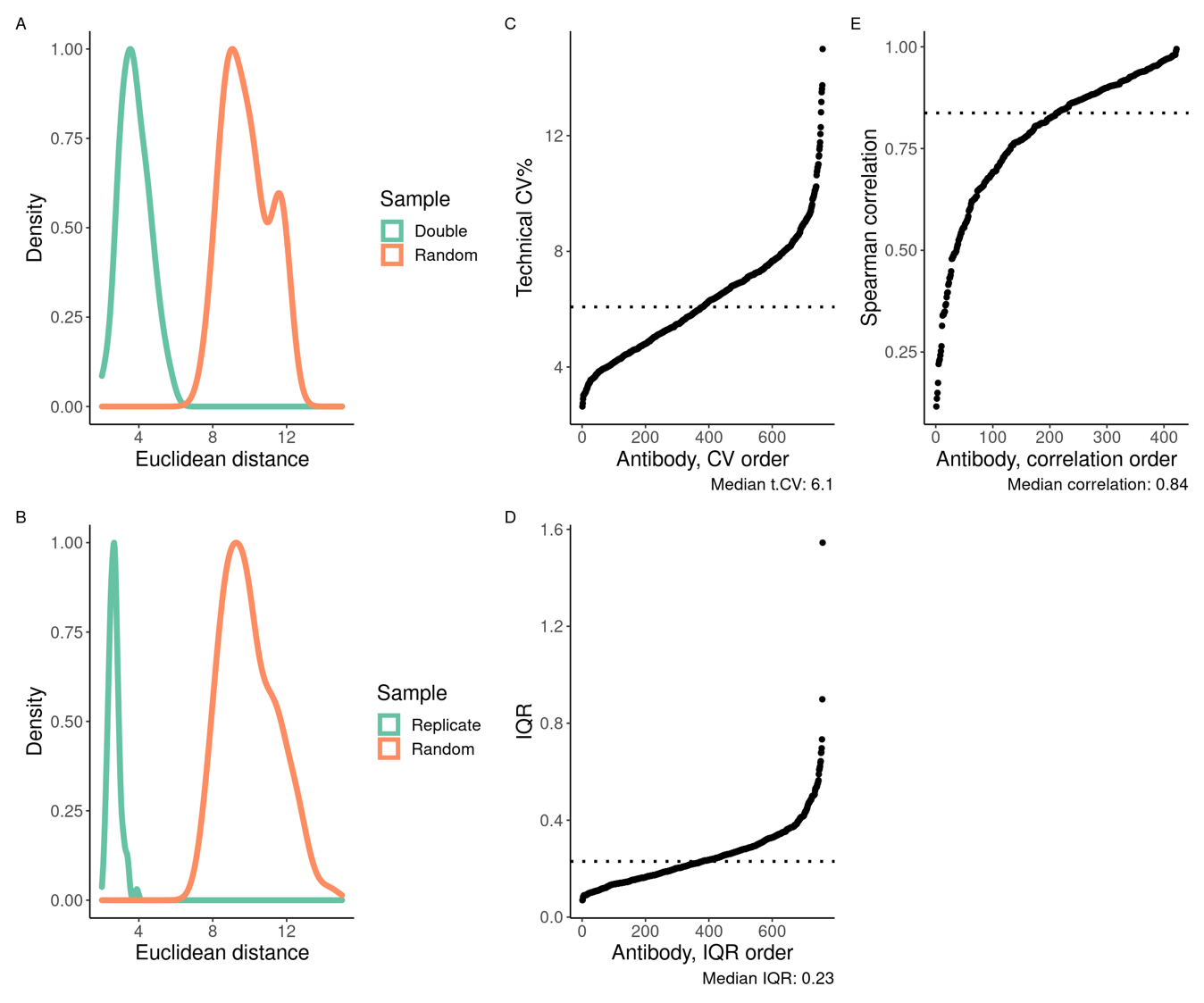


***Figure S15****: QC plots: Euclidean distances (normalized, log-transformed, and antibody-filtered data) between (A) 30 pairs of doubles and an equal number of pairs of random samples, (B) 94 pairs of replicated samples, and an equal number of pairs of random samples, (C) technical CV (based on pooled samples) of antibodies, the median is 6.1%, (D) interquartile range (IQR) of normalized and log-transformed data for antibodies remaining after filtering (median IQR: 0.23), and (E) Spearman’s correlation for antibodies used in repeated assays using the same antibody array, median correlation is rho=0.84.*
